# Assessing Patient Satisfaction with Hospital Services: Perspectives from Bihor County Emergency Hospital, Romania

**DOI:** 10.3390/healthcare13070836

**Published:** 2025-04-07

**Authors:** Aliz Ildiko Bradács, Florica Voiță-Mekeres, Lucia Georgeta Daina, Lavinia Davidescu, Călin Tudor Hozan

**Affiliations:** 1 Doctoral School of Biomedical Sciences, Faculty of Medicine and Pharmacy, University of Oradea, 410087 Oradea, Romania; aliz.bradacs@uoradea.ro (A.I.B.); lgdaina@uoradea.ro (L.G.D.); 2 Department of Morphological Disciplines, Faculty of Medicine and Pharmacy, University of Oradea, 410087 Oradea, Romania; 3 Department of Psycho-Neuroscience and Rehabilitation, University of Oradea, 410073 Oradea, Romania; 4 Department of Medical Disciplines, Faculty of Medicine and Pharmacy, University of Oradea, 410087 Oradea, Romania; lavinia.davidescu@didactic.uoradea.ro; 5 Department of Surgical Disciplines, Faculty of Medicine and Pharmacy, University of Oradea, 410087 Oradea, Romania; chozan@uoradea.ro

**Keywords:** patient satisfaction, hospital services, continuity of care, healthcare quality, patient experience

## Abstract

Background/Objectives: The objective of this study is to assess overall patient satisfaction with hospital services, including cleanliness, ward conditions, and food quality. Another key goal is to determine patient willingness to return for future medical services and identify the factors influencing this decision. Moreover, the study explores the relationship between patient satisfaction and continuity of care, as indicated by previous hospitalizations. Methods: We conducted a retrospective cohort study to evaluate patient satisfaction at the Bihor County Emergency Clinical Hospital in Oradea, Romania. A standardized 40-item questionnaire was developed in accordance with the Framework Agreement on the provision of medical assistance within the Romanian healthcare system. The survey, which was administered over a four-year period (2019–2022), covered seven domains: demographic data, accessibility, hotel conditions, quality of care, patient safety and rights, overall satisfaction, and feedback. A total of 12,802 patients completed the questionnaire, and all statistical analyses were performed using R Studio. Results: This study analyzes patient-reported satisfaction and experiences in a large healthcare facility, based on data from 12,802 participants. Overall, 91% of respondents rated the hospital positively, with 62% giving an excellent score. Spiritual assistance was well received (71%), and 70% of patients expressed willingness to return for future medical needs. Hospital cleanliness and ward conditions were rated highly, with 71% of respondents reporting excellent experiences. Food quality was positively reviewed by 66% of participants. Most patients (95%) confirmed proper hygiene practices by medical staff, and 95% were informed about their diagnosis. However, only 67% were aware of the complaint submission process. The dataset spans 2019–2022, with the highest hospitalizations in 2020 (36%) and obstetrics, cardiology, and general surgery being the most common specialties. Conclusions: This dataset reflects a high level of patient satisfaction across multiple dimensions of hospital services, including cleanliness, quality of care, and patient information. However, areas such as complaint handling and transparency in medication handling require further attention to improve the overall patient experience. The findings underscore the hospital’s strong performance in meeting patient expectations while identifying key areas for continued improvement.

## 1. Introduction

Patient satisfaction is a fundamental measure of healthcare quality that reflects the effectiveness, safety, and patient-centeredness of medical services. It not only shapes a hospital’s reputation but also influences patient adherence to treatment, clinical outcomes, and overall trust in healthcare providers. High levels of patient satisfaction are associated with improved health outcomes, increased patient retention, and fewer legal or ethical issues, whereas dissatisfaction can lead to reduced compliance and negative perceptions of care [1,2,3,4,5]. Despite its widespread use as a quality indicator, patient satisfaction is a multifaceted construct that requires careful interpretation; for example, satisfied patients may still report specific areas of dissatisfaction, and survey biases—particularly in low- and middle-income settings—can lead to overestimations of satisfaction levels [6,7]. Assessing patient satisfaction involves examining multiple dimensions of hospital care, including the quality of medical treatment, staff professionalism, communication effectiveness, waiting times, and the overall patient experience from admission to discharge. The growing emphasis on patient-centered care has prompted healthcare organizations to integrate patient feedback into quality improvement strategies [2,4,8,9].

In this context, both clinical outcomes and service performance indicators play critical roles. For instance, treatment outcomes and nursing kindness have been shown to be strong predictors of patient satisfaction [10,11], while organizational factors—such as nurse-to-patient ratios and the hospital work environment—also significantly impact patients’ ratings and recommendations [12]. Moreover, individual patient characteristics, including confidence in the local healthcare system and overall life satisfaction, may have a greater influence on satisfaction than traditional demographic factors [13,14]. Recent evidence highlights the importance of patient satisfaction in driving better health outcomes. In diabetes care, for example, higher satisfaction across domains such as continuity of care and trust has been correlated with improved glycemic control [15]. Similarly, in HIV care, greater satisfaction with health services has been associated with improved adherence to antiretroviral therapy [16]. Trust in healthcare providers, which is closely linked to satisfaction, further reinforces these positive outcomes, particularly among older adults [17]. The COVID-19 pandemic and the subsequent rise in telehealth services have also influenced patient satisfaction, with many patients reporting high levels of satisfaction with remote consultations despite some overall declines in consumer loyalty [18,19]. Patient satisfaction in hospital settings is influenced by a wide array of factors, including institutional characteristics, geographical and cultural context, and individual patient expectations. Research indicates that aspects such as the number of healthcare professionals per bed, the size of the institution, and treatment toxicity can affect satisfaction levels. Moreover, different hospital staff groups have distinct expectations, which in turn can indirectly influence patient satisfaction [20,21]. Over time, targeted interventions—such as reducing waiting times and incorporating new technologies like robotic-assisted surgeries—have shown promise in improving satisfaction rates [22,23]. The rapid expansion of telehealth services during the pandemic has further demonstrated that patient satisfaction is an evolving metric, responsive to changes in healthcare delivery models [24,25].

Conversely, patient dissatisfaction carries significant implications for healthcare delivery and outcomes. Dissatisfied patients are less likely to adhere to treatment plans and may experience poorer health outcomes, as observed in conditions like asthma [26]. Such dissatisfaction can also lead to a reduction in the utilization of services and may even escalate to conflicts between patients and providers [27]. These dynamics underscore the need for healthcare systems to address underlying causes of dissatisfaction to improve overall care quality.

This study provides a comprehensive analysis of patient-reported satisfaction at the Bihor County Emergency Clinical Hospital, based on data collected from 12,802 participants over a four-year period (2019–2022). By examining key aspects of medical care, staff interactions, hospital cleanliness, and transparency in communication, the study aims to identify both the strengths and challenges of hospital service delivery. The findings are intended to guide quality improvement initiatives and enhance patient-centered care, with particular attention to the evolving healthcare landscape in Eastern Europe.

## 2. Materials and Methods

### 2.1. Study Design 

The study is a retrospective cohort study covering the period 2019–2022 and was carried out at the County Clinical Emergency Hospital Oradea (CCEHO) by analyzing patient satisfaction questionnaires. The CCEHO is a tertiary-level public hospital located in northwestern Romania, which provides medical assistance to approximately 200,000 inhabitants of the Municipality of Oradea and emergency medical services to a territorial population of around 600,000 inhabitants. The average number of patients discharged annually is about 40,000, and approximately 3200 questionnaires are applied each year, representing an average of about 8.00% of the discharged patients who fully responded to the administered questionnaires. The evaluation of patient satisfaction is part of the implementation of a unitary/standardized monitoring mechanism for performance in health facilities, aimed at reducing practice variability, focusing on quality, and providing feedback for quality improvement [28].

### 2.2. Data Collection and Tools

A standardized questionnaire comprising 40 questions was developed in accordance with the monitoring obligations of patient satisfaction as stipulated by the Framework Agreement concerning the conditions for the provision of medical assistance within the Romanian healthcare system [29]. The questionnaire is available in the Appendix A. The questionnaire is divided into seven sections or domains, each containing between 1 and 15 questions. These domains encompass demographic data, accessibility/admission, hotel conditions, quality of medical care, patient safety and rights, overall satisfaction, and observations/suggestions. The study was based on cross-sectional surveys administered to patients at the time of discharge; no subsequent follow-up was conducted after the initial survey. This approach was chosen to capture immediate patient perceptions of hospital services. The questionnaire was administered to all patients discharged from the hospital during the study period as part of routine quality monitoring. Inclusion criteria required that respondents be adults who had received inpatient care at CCEHO, ensuring that the data reflected a wide cross-section of the hospital’s patient population.

### 2.3. Statistical Analysis

All statistical analyses were performed using R version 4.4.3, developed by the R Core Team and sourced from the R Foundation for Statistical Computing in Vienna, Austria. Continuous variables were summarized using means and standard deviations or medians and interquartile ranges (IQRs), as appropriate, after testing for normality with the Shapiro–Wilk test. Categorical variables were described using frequencies and percentages. Comparisons of categorical data across different years were performed using chi-square tests to assess statistical significance, with *p* values less than 0.05 considered significant. To account for multiple comparisons, a Bonferroni correction was applied, which adjusted the significance threshold accordingly. The analysis also included trend evaluations of key indicators over the four-year study period (see supplementary Table S1).

For further clarity in data presentation, Likert-scale items were analyzed both individually and by grouping responses into positive, neutral, and negative categories. For example, responses in Ratings 4 and 5 were classified as positive, responses in Ratings 1 and 2 as negative, and Rating 3 as neutral. This categorization facilitated the examination of temporal trends and allowed for comparisons across different dimensions of patient satisfaction. For the quality of medical service domain, Cronbach’s alpha was 0.686, and for the hotel condition domain, it was 0.632. These estimates were obtained by performing a reliability analysis solely on items measured with a 1–6 Likert scale, thereby excluding items with different response formats (e.g., yes/no/no response). Although both values are slightly below the conventional 0.70 threshold for acceptable reliability, they indicate moderate internal consistency, which can be considered reasonable in exploratory research or when scales contain a limited number of items.

The study was conducted in accordance with the Declaration of Helsinki and approved by the Institutional Review Board (or Ethics Committee) of County Clinical Emergency Hospital Oradea, Bihor, Romania (9406. 08.04.2021 and 9391.07.04.2021).

## 3. Results

The dataset provides a comprehensive analysis of patient-reported satisfaction and experience in a large healthcare facility, comprising 12,802 participants. The study included 5310 male patients (41.5%). Obstetrics accounted for 17.7% of admissions (2262 patients overall), cardiology for 12.2% (1560 patients overall), and general surgery for 12.1% (1548 patients overall). Other specialties included the burn unit at 5.8% (746 patients) and ENT at 4.2% (542 patients). A chi-square test indicated that these differences were statistically significant (*p* < 0.001).

**Figure 1:**
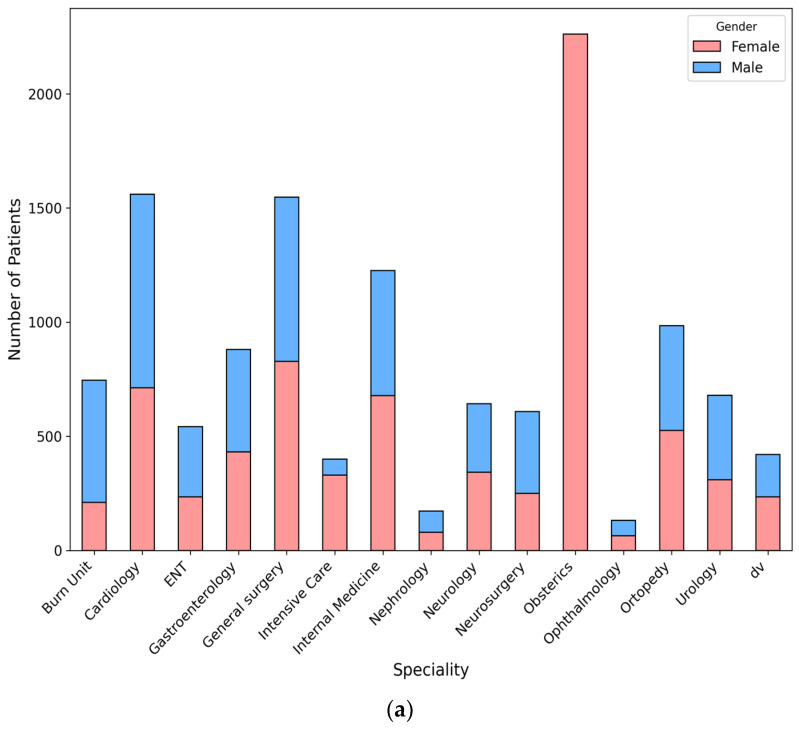

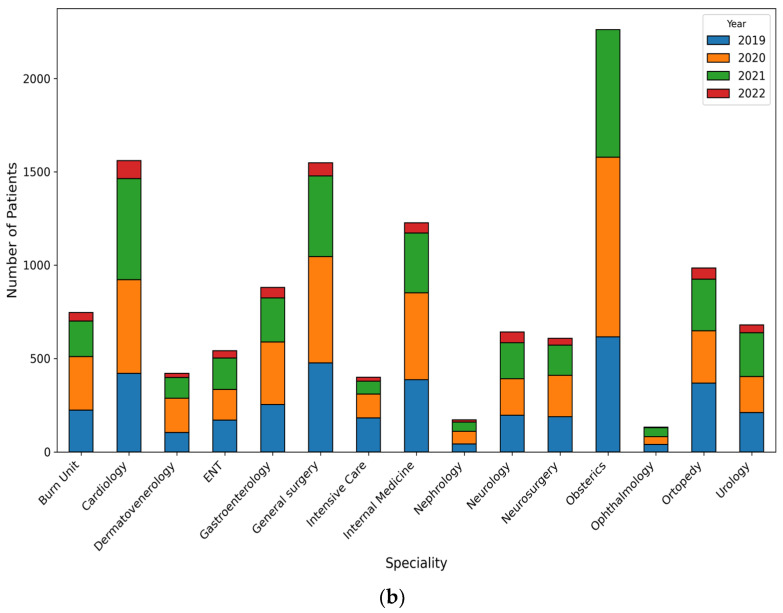
(**a**) Distribution of specialties by gender. (**b**) Distribution of specialties by year.

The distribution of patient admissions by specialty varied significantly by gender (*p* < 0.001) For example, cardiology accounted for 9.5% of admissions among females compared to 16.0% among males; similarly, neurosurgery represented 3.3% of female admissions versus 6.7% of male admissions, and intensive care accounted for 4.4% of female admissions compared to 1.3% of male admissions. These gender-based differences, along with variations in other specialties such as general surgery (11.1% for females versus 13.6% for males), indicated statistically significant disparities in specialty utilization (Figure 1).

Educational background varied among participants, with 50% reporting high school as their highest level of education, followed by 24% with a university degree. Regarding residence, 43% lived in urban areas, 40% in rural areas, and 16% did not specify (Table 1). 

The overall impression of the hospital was overwhelmingly positive, with 62% of respondents rating it as excellent (5/5) and 29% as very good (4/5). A small minority rated their experience as poor (1/5: 1.2%) or fair (2/5: 0.6%). Satisfaction with the spiritual assistance provided by the hospital was also high, with 71% responding affirmatively, although 26% did not provide an answer.

A significant proportion of respondents (70%) indicated a willingness to return to the hospital if medical services were required in the future, while 18% expressed uncertainty, and 2% stated they would not return. Furthermore, 57% of participants reported prior hospitalizations in the same facility, indicating a degree of continuity in care for many patients.

Regarding the quality of the hospital’s sanitary facilities, 46% of participants rated them as excellent, with 32% rating them as very good. Cleanliness within patient rooms was rated as excellent by 71% of respondents, with the majority (67%) reporting that their rooms were cleaned as needed.

When asked about ward conditions, 55% of respondents rated the facilities and equipment as excellent, and 30% as very good. Hospital food quality was also positively evaluated, with 34% rating it as excellent and 32% as very good, although 8.3% refrained from responding.

Of the patients surveyed, 85% reported not rewarding medical staff with money or gifts, while 4.7% admitted to doing so. Among those who rewarded staff, 8.2% specified nurses as recipients, while 1.6% cited doctors.

Postoperative care and services provided in the intensive care unit, when applicable, were rated highly, with 36% giving an excellent score and 6.3% rating it as very good. Among participants, 43% underwent surgery during their hospitalization.

A substantial majority (95%) stated that medical staff consistently used disposable gloves during patient contact. Similarly, 71% confirmed that medication vials were opened in their presence. However, 9.2% reported that this standard was not followed.

**Figure 2:**
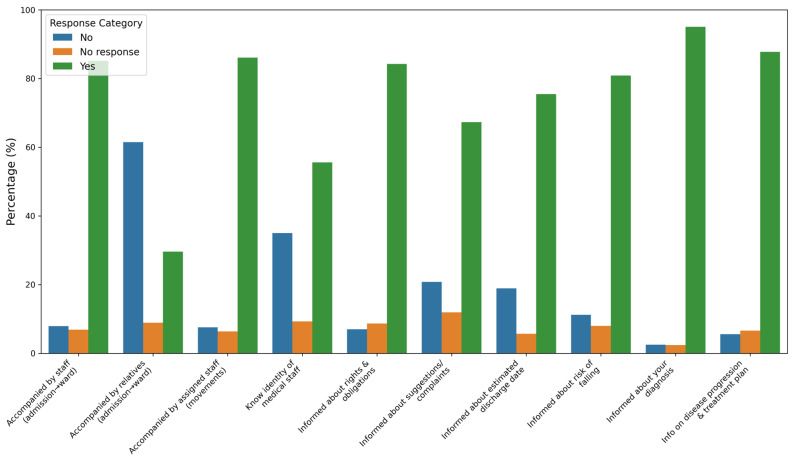
Distribution of patient responses regarding the process for submitting suggestions and complaints.

When asked whether they were informed about their medical diagnosis, 95% responded affirmatively, with a similarly high proportion (88%) stating they received adequate information about their disease progression and treatment plans. Awareness of the risk of falling and the estimated discharge date was reported by 81% and 75% of participants, respectively. However, fewer respondents (67%) reported being informed about the process for submitting complaints and suggestions (Figure 2). 

Specialty-wise, the majority of patients were admitted to the obstetrics department (18%), followed by cardiology (12%) and general surgery (12%). Other specialties such as orthopedics (7.7%), gastroenterology (6.9%), and internal medicine (9.6%) accounted for significant proportions of admissions.

**Figure 3:**
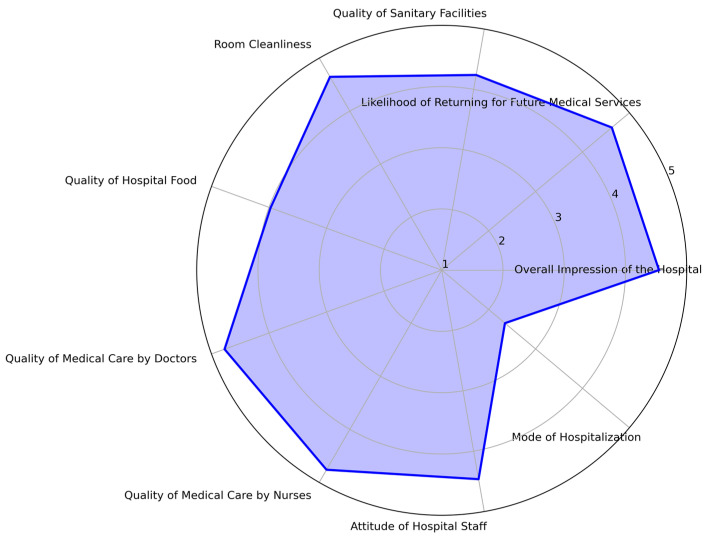
Radar chart of patient satisfaction dimensions across hospital services.

The data spans a four-year period from 2019 to 2022, with most patients hospitalized in 2020 (36%) and 2019 (30%). Seasonal variations were observed, with the first trimester accounting for 29% of hospitalizations, closely followed by the third trimester (27%). Figure 3 provides a radar chart depicting key patient satisfaction dimensions across the hospital’s services from 2019 to 2022.

In terms of hospitalization methods, the majority of patients presented directly to the emergency room (35.9%), followed by ambulance arrivals (26.4%). Referrals by family doctors decreased over time, from 27.7% in 2019 to 16.9% in 2022, while referrals from outpatient doctors remained relatively stable at approximately 8%. These differences were statistically significant (*p* < 0.001).

Accompaniment by medical staff from the admission office to the ward increased over the years, with 77.8% reporting being accompanied in 2019 and 91.4% in 2021, stabilizing at 88.3% in 2022. In contrast, accompaniment by relatives declined sharply, from 49.4% in 2019 to 13.6% in 2022, indicating a shift in patient handling procedures. Both trends were highly significant (*p* < 0.001).

Patients’ knowledge of the identity of medical staff remained consistent, with approximately 55% of respondents affirming familiarity. However, the proportion of those unaware hovered at 35%, while about 9% provided no response. Similarly, ratings for the attitude of hospital staff revealed that 68.5% rated it as excellent (5/5), with consistent improvements observed from 2019 to 2022 (*p* < 0.001).

**Figure 4:**
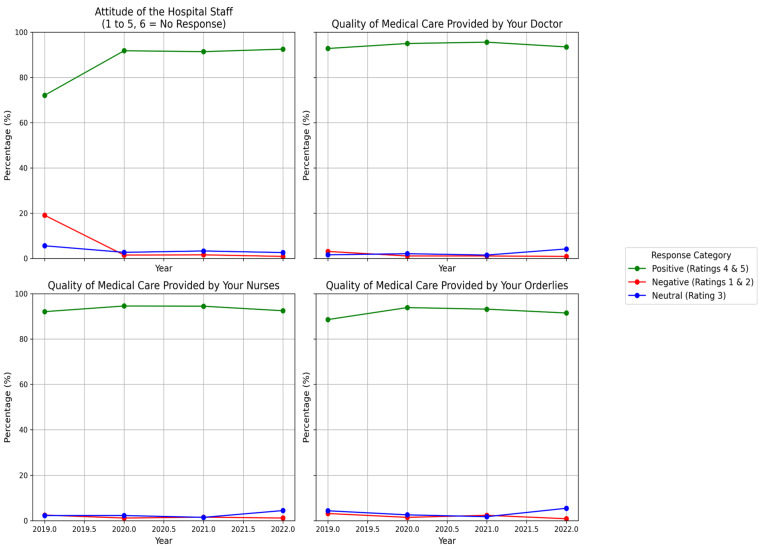
Trends in positive, negative, and neutral ratings for four key aspects of patient satisfaction over time.

When evaluating the quality of care by medical professionals, doctors earned the highest satisfaction, with 82.2% rating the quality of care as excellent (5/5). Nurses followed closely at 81.1%, while orderlies were rated slightly lower at 77.9%. This stratification highlights the high overall quality of care across staff categories (*p* < 0.001) (Figure 4).

Regarding information dissemination, 95.1% of patients reported being informed about their diagnosis, while 87.8% were informed about disease progression and treatment plans. Awareness of the risk of falling (80.9%) and the estimated discharge date (75.5%) was comparatively lower but showed improvement across the study period (*p* < 0.001). Information regarding the submission of complaints and suggestions remained an area requiring attention, with only 67.3% affirming they were informed about the process.

Sanitary and ward conditions received high satisfaction ratings. For sanitary facilities, 45.5% rated them as excellent, while 71.3% rated room cleanliness as excellent. Additionally, 55.4% expressed high satisfaction with ward conditions, reflecting a well-maintained hospital environment.

**Figure 5:**
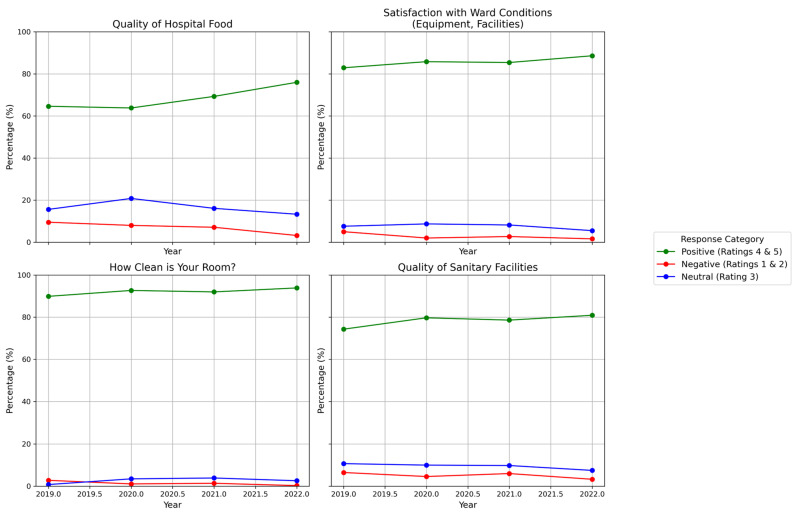
Trends in positive, negative, and neutral ratings for four aspects of the hospital environment.

Hospital food quality received mixed responses, with 34.2% rating it as excellent, while 17.5% found it average (3/5) (Figure 5).

Spiritual assistance satisfaction was reported by 71% of respondents, with no significant yearly variations. Overall impressions of the hospital remained positive, with 61.9% rating it as excellent and 28.6% as very good (4/5), demonstrating sustained patient satisfaction.

Procedural and safety aspects were also well regarded. A significant majority (95.3%) stated that disposable gloves were used during every interaction, while 71% reported that medication vials were opened in their presence. However, 9.2% noted that vials were not opened in their presence, which indicates room for improvement in maintaining transparency.

The data also reveals trends in financial interactions with hospital staff. While 84.6% of patients reported not rewarding staff with money or gifts, 4.7% admitted to doing so. Doctors and nurses were the primary recipients.

Table 2 summarizes changes in hospitalization-seeking behavior and support practices from 2019 to 2022 (N = 12,802), with all overall comparisons reaching significance (*p* < 0.001). Patients arriving by ambulance increased from 826 (21.3%) in 2019 to 200 (32.5%) in 2022 (3376 overall, 26.4%), while those presenting directly to the emergency room went from 1166 (30.1%) in 2019 to 196 (31.8%) in 2022 (4600 overall, 35.9%). Referrals by family doctors declined from 1,074 (27.7%) in 2019 to 104 (16.9%) in 2022 (2278 overall, 17.8%), and other categories showed minor yet statistically significant variations. Pairwise comparisons for this variable were all significant, with *p*-values as follows: 2021 vs. 2020 (<0.000001), 2021 vs. 2019 (<0.000001), 2021 vs. 2022 (0.000011), 2020 vs. 2019 (<0.000001), 2020 vs. 2022 (0.000326), and 2019 vs. 2022 (<0.000001).

Accompaniment by medical staff improved from 3014 (77.8%) in 2019 to 544 (88.3%) in 2022 (10,910 overall, 85.2%), with unaccompanied patients dropping from 594 (15.3%) to 28 (4.5%). Significant differences were observed between 2021 vs. 2020 (<0.000001), 2021 vs. 2019 (<0.000001), and 2019 vs. 2022 (<0.0001), while 2021 vs. 2022 (0.029418) and 2020 vs. 2022 (0.225031) did not reach significance after Bonferroni correction.

In contrast, accompaniment by relatives decreased sharply from 1916 (49.4%) in 2019 to 84 (13.6%) in 2022 (3792 overall, 29.6%), with unaccompanied cases rising from 1608 (41.5%) to 490 (79.5%). Here, pairwise differences were significant for 2021 vs. 2020 (<0.000001), 2021 vs. 2019 (<0.000001), 2020 vs. 2019 (<0.000001), 2020 vs. 2022 (<0.000001), and 2019 vs. 2022 (<0.000001), while the difference for 2021 vs. 2022 (0.277169) was not.

Accompaniment by assigned staff during hospital movements increased from 3172 (81.8%) in 2019 to 550 (89.3%) in 2022 (11,018 overall, 86.1%), with unaccompanied patients declining from 412 (10.6%) to 38 (6.2%). Significant differences were found for 2021 vs. 2020 (0.000005), 2021 vs. 2019 (<0.000001), 2020 vs. 2019 (<0.000001), and 2019 vs. 2022 (0.000031), whereas 2021 vs. 2022 (0.525754) and 2020 vs. 2022 (0.075726) were not significant.

For people asked about the identity of the staff (overall *p* < 0.0011), the proportion affirming knowledge declined from 57.5% in 2019 to 53.9% in 2022, while those not knowing increased from 33.6% to 40.9%. Pairwise comparisons showed significant differences between 2021 and 2022 (adjusted *p* = 0.0002), 2020 and 2022 (adjusted *p* = 0.000113), and 2019 and 2022 (adjusted *p* = 0.00012); however, differences between 2021 and 2020 (*p* = 0.916925), 2021 and 2019 (*p* = 0.044746), and 2020 and 2019 (*p* = 0.043793) did not meet the Bonferroni-adjusted threshold of *p* < 0.00833. For ratings of hospital staff attitude (overall *p* < 0.0011), the top rating of 5 increased from 59.0% in 2019 to 75.0% in 2022, and the lowest rating fell from 18.5% to 0.6%. Significant differences were found between 2021 and 2020 (adjusted *p* = 0.001), 2021 and 2019 (adjusted *p* < 0.0001), 2020 and 2019 (adjusted *p* < 0.0001), and 2019 and 2022 (adjusted *p* < 0.000001), whereas comparisons between 2021 and 2022 (*p* = 0.777819) and 2020 and 2022 (*p* = 0.338907) were not significant. For the quality of care provided by doctors (overall *p* < 0.0011), with top ratings of 83.3% in 2019, 81.7% in 2020, 82.0% in 2021, and 81.2% in 2022 (overall 82.2%), significant differences emerged between 2021 and 2019 (adjusted *p* = 0.0001), 2021 and 2022 (adjusted *p* = 0.00027), 2020 and 2019 (adjusted *p* < 0.001), and 2019 and 2022 (adjusted *p* = 0.0001), while differences between 2021 and 2020 (*p* = 0.305) and 2020 and 2022 (*p* = 0.0285) were not significant. For the quality of care provided by nurses (overall *p* < 0.0011), with top ratings of 81.4% in 2019, 80.6% in 2020, 81.5% in 2021, and 81.8% in 2022 (overall 81.1%), all pairwise comparisons were significant: 2021 vs. 2020 (adjusted *p* = 0.00398), 2021 vs. 2019 (adjusted *p* = 0.0000154), 2021 vs. 2022 (adjusted *p* = 0.0001), 2020 vs. 2019 (adjusted *p* < 0.001), 2020 vs. 2022 (adjusted *p* = 0.00591), and 2019 vs. 2022 (adjusted *p* = 0.0001). Finally, for the quality of care provided by orderlies (overall *p* < 0.0011), with top ratings of 77.1% in 2019, 77.9% in 2020, 78.8% in 2021, and 77.9% in 2022 (overall 77.9%), significant differences were observed between 2021 and 2020 (adjusted *p* = 0.000799), 2021 and 2019 (adjusted *p* < 0.001), 2021 and 2022 (adjusted *p* = 0.0001), 2020 and 2022 (adjusted *p* = 0.00138), and 2019 and 2022 (adjusted *p* = 0.001), all meeting the Bonferroni-corrected threshold of *p* < 0.00833 (Table 3).

Over the years, 53.8% of patients were informed about their rights and obligations at the admission office (30.5% received verbal information, and 7.0% were not informed), with significant overall differences (*p* < 0.0011). Pairwise comparisons showed that differences between 2021 and 2020 (adjusted *p* = 0.00214), between 2021 and 2019 (adjusted *p* = 0.0000000000472), between 2021 and 2022 (adjusted *p* = 0.00618), and between 2020 and 2022 (adjusted *p* = 0.0000478) were significant, whereas the difference between 2019 and 2022 (adjusted *p* = 0.0192) was not.

Information about how to submit suggestions and complaints was provided to 67.3% of patients (overall *p* < 0.0011); here, only the comparisons between 2021 and 2019 (adjusted *p* = 0.000000556) and between 2020 and 2019 (adjusted *p* = 0.000121) reached significance. Seventy-five and a half percent of patients were informed about their estimated discharge date (overall *p* < 0.0011), with significant differences only between 2021 and 2019 (adjusted *p* = 0.000015) and between 2020 and 2019 (adjusted *p* = 0.000025); differences between 2021 and 2020, 2021 and 2022, and 2020 and 2022 did not reach significance.

Information on the risk of falling was provided to 80.9% of patients (overall *p* < 0.0011); significant differences were seen between 2021 and 2019, between 2020 and 2019, and between 2019 and 2022 (all adjusted *p* < 0.001). Nearly all patients (95.1%) were informed about their diagnosis (overall *p* < 0.0011), with significant differences only between 2021 and 2019 and between 2020 and 2019 (adjusted *p* < 0.001). Information on disease progression and treatment plans was provided to 87.8% of patients (overall *p* < 0.0011), with significant differences between 2021 and 2020, between 2021 and 2019, and between 2020 and 2019 (all adjusted *p* < 0.001), while other comparisons were not significant.

Seventy-three percent of patients received information on medication side effects (overall *p* < 0.0011); significant differences were observed between 2021 and 2019, between 2021 and 2022, between 2020 and 2019, between 2020 and 2022, and between 2019 and 2022 (all adjusted *p* < 0.001), whereas the difference between 2021 and 2020 (adjusted *p* = 0.04108) was not significant. Only 35.0% of patients could name a medication administered to them (overall *p* < 0.0011); here, significant differences were found between 2021 and 2019, between 2021 and 2022, and between 2020 and both 2019 and 2022 (all adjusted *p* < 0.001), while the difference between 2019 and 2022 (adjusted *p* = 0.06308) was not significant.

Details on purchased medications were documented in 87.0% of cases (overall *p* = 0.0151), but none of the pairwise comparisons reached significance. Seventy-one percent of patients reported that vials were opened in their presence, but overall differences were not significant (*p* = 0.1901). Disposable glove use was nearly universal (95.3% overall) with overall significance (*p* = 0.0021); a significant difference was observed only between 2020 and 2019 (adjusted *p* = 0.00135).

For operations during hospitalization, overall differences were not significant (*p* = 0.0781) except between 2020 and 2019 (adjusted *p* = 0.00603). Postoperative or intensive care ratings, available from 36.4% of respondents, and hospital food quality ratings (with 34.2% awarding the top score) both showed overall significant differences (*p* < 0.0011), with all pairwise comparisons for food quality significant. Finally, ward condition satisfaction was high (55.4% giving the top score) with significant differences between 2021 and 2019, between 2020 and 2019, and between 2019 and 2022 (all adjusted *p* < 0.001), and rewarding of medical staff was uncommon (84.6% did not reward), with all pairwise comparisons for this variable showing adjusted *p*-values below the Bonferroni-corrected threshold (Table 4).

Table 5 shows that 71.3% of respondents rated their room cleanliness with a top score of 5 and only 1.1% gave a score of 1, with highly significant overall differences (*p* < 0.0011). In pairwise comparisons, differences between 2021 and 2019 and between 2020 and 2019 were significant (adjusted *p* < 0.001), as was the difference between 2019 and 2022 (adjusted *p* = 0.000228), while comparisons between 2021 and 2020 (*p* = 0.333), between 2021 and 2022 (*p* = 0.0931), and between 2020 and 2022 (*p* = 0.261) were not significant. In total, 66.7% of patients reported that their room was cleaned “as needed,” and 23.8% indicated cleaning twice daily; overall differences in cleaning frequency were significant (*p* < 0.0011). Significant pairwise differences were found between 2021 and 2022 (adjusted *p* = 0.000158), between 2020 and 2022 (adjusted *p* = 0.00000025), and between 2019 and 2022 (adjusted *p* = 0.000011), while the other comparisons did not reach significance. For the quality of sanitary facilities, 45.5% of respondents gave a top score and 32.3% rated them as 4; overall, this variable differed significantly across years (*p* < 0.0011). Pairwise comparisons revealed significant differences between 2021 and 2019 (adjusted *p* < 0.001), between 2021 and 2022 (adjusted *p* = 0.00142), between 2020 and 2019 (adjusted *p* < 0.001), and between 2019 and 2022 (adjusted *p* = 0.000204), while the differences between 2021 and 2020 (*p* = 0.0221) and between 2020 and 2022 (*p* = 0.0209) were not significant. Finally, 77.5% of patients reported that visitation rules were respected and only 4.0% disagreed; overall differences were significant (*p* < 0.0011). For visitation rules, significant differences emerged between 2021 and 2020, between 2021 and 2019, between 2020 and 2019, between 2020 and 2022, and between 2019 and 2022 (all adjusted *p* < 0.001), while the comparison between 2021 and 2022 (*p* = 0.235) was not significant (Table 5).

Across the years, 58.9% of respondents in 2019, 56.7% in 2020, 57.2% in 2021, and 54.5% in 2022 reported previous hospitalization, yielding an overall rate of 57.4% (*p* < 0.0011). Pairwise comparisons for previous hospitalization showed no significant differences between 2021 and 2020 (adjusted *p* = 0.101) or between 2021 and 2019 (adjusted *p* = 0.0378), but significant differences emerged when comparing 2021 with 2022 (adjusted *p* = 0.0000000751), 2020 with 2022 (adjusted *p* = 0.0000612), and 2019 with 2022 (adjusted *p* = 0.0000151).

When asked about the likelihood of returning for future medical services, 72.5% in 2019, 69.6% in 2020, 68.5% in 2021, and 70.8% in 2022 selected the highest rating, resulting in an overall positive response of 70.2% (*p* < 0.0011). Here, significant differences were found between 2021 and 2020 (adjusted *p* = 0.000321), between 2021 and 2019 (adjusted *p* = 0.0000000378), and between 2020 and 2019 (adjusted *p* = 0.00000032), while the comparisons between 2021 and 2022 (adjusted *p* = 0.266), between 2020 and 2022 (adjusted *p* = 0.0165), and between 2019 and 2022 (adjusted *p* = 0.105) were not significant.

Regarding satisfaction with spiritual assistance, 72.7% of respondents in 2019, 71.3% in 2020, 69.7% in 2021, and 66.6% in 2022 expressed satisfaction, giving an overall rating of 71.0% (*p* < 0.0011). Pairwise comparisons indicated significant differences between 2021 and 2019 (adjusted *p* = 0.000165), between 2021 and 2022 (adjusted *p* = 0.002978), between 2020 and 2022 (adjusted *p* = 0.001943), and between 2019 and 2022 (adjusted *p* = 0.002302), while comparisons between 2021 and 2020 (adjusted *p* = 0.131) and between 2020 and 2019 (adjusted *p* = 0.0545) were not significant.

For the overall impression of the hospital, 64.4% of respondents in 2019, 60.4% in 2020, 61.0% in 2021, and 63.0% in 2022 awarded the highest rating, corresponding to an overall positive impression of 61.9% (*p* < 0.0011). Significant differences were observed between 2021 and 2019 (adjusted *p* = 0.000000164) and between 2020 and 2019 (adjusted *p* = 0.000000535), while the comparisons between 2021 and 2020 (adjusted *p* = 0.126), between 2021 and 2022 (adjusted *p* = 0.306), and between 2020 and 2022 (adjusted *p* = 0.381) were not significant, nor was the difference between 2019 and 2022 (adjusted *p* = 0.014) (Table 6).

This study reflects a high level of patient satisfaction across multiple dimensions of hospital services, including cleanliness, quality of care, and patient information. However, areas such as complaint handling and transparency in medication handling require further attention to improve the overall patient experience. The findings underscore the hospital’s strong performance in meeting patient expectations while identifying key areas for continued improvement.

## 4. Discussion

The findings show notable variations across the years in terms of how patients accessed the hospital, the level of assistance provided, and the information received during their hospitalization.

The analysis of the survey responses across four years (2019−2022) from a total of 12,802 patients provides valuable insights into hospital services and patient satisfaction. 

One of the most striking trends observed in the data is the increased reliance on ambulance transport and direct emergency room visits over the years. In 2019, 21.3% of patients arrived by ambulance, which increased to 32.5% in 2022. This shift may reflect a growing awareness among patients regarding the severity of their conditions or improvements in ambulance services and their response times. Similarly, the number of patients presenting directly to the emergency room has steadily increased, with a notable rise from 30.1% in 2019 to 40.2% in 2021. 

A retrospective study was performed based on satisfaction questionnaires addressed to patients hospitalized in the Orthopedics and Traumatology departments of the County Clinical Emergency Hospital Oradea between 2015 and 2019. The results indicate that overall patient satisfaction—or the general impression of the hospital—is strongly dependent on the quality of medical care provided by the doctors as well as the specific hotel conditions of the facility [30]. This observation aligns with broader trends in the healthcare industry, where increased emergency room utilization is often linked to growing patient trust in emergency services and a greater willingness to seek care for acute conditions [31]. Furthermore, the increasing reliance on ambulance transport and emergency room visits can be attributed to several factors. Population aging is a significant contributor to the rising demand for emergency medical services (EMSs); as the proportion of older adults increases, there is a higher incidence of falls and other age-related health issues requiring emergency care [12,32].

For instance, among people aged ≥ 65 years, falling is the leading cause of emergency department visits, accounting for 17% of all EMS calls [32]. Changes in social support structures, increased accessibility and pricing of ambulance services, and growing community health awareness have also been proposed as factors driving higher utilization [33]. Additionally, the prevalence of mental health issues, alcohol/drug abuse, and patients with high comorbidity scores has led to significant annual growth in ambulance demand [34]. Interestingly, there are contradictions in the data regarding age-specific incidence rates. While Andrew et al. (2020) [34] reported significant increases in ambulance use for patients aged <60 years, Hanchate et al. [35] indicated higher rates among those aged ≥85 years. Furthermore, a substantial proportion of ambulance transport (21%) did not result in hospital admission, suggesting potential overuse of emergency services [32]. 

In contrast, there has been a gradual decline in the number of patients referred by family doctors, which decreased from 27.7% in 2019 to 16.9% in 2022. This reduction, especially in the urban areas compared to rural areas, may be indicative of evolving referral practices, changes in primary care access, or a shift toward self-referral through emergency services [36]. The role of primary care referrals has evolved significantly in recent years, with several key trends emerging: Primary care doctoring in the USA has undergone substantial changes, with some experts predicting it could become rare or even nonexistent by 2025 [37]. Factors contributing to this shift include the epidemiological transition to chronic diseases, overcrowding in healthcare, and the rise in non-physician clinicians. Many everyday illnesses may be managed via the Internet or by non-physician clinicians in retail clinics, with specialists handling more complex cases rather than primary care doctors [37]. Interestingly, the definition and measurement of primary care have also evolved. Research has shown that specialties beyond family/general practice, pediatrics, and internal medicine make significant contributions to primary care [38]. The role of primary care referrals has become more complex, with challenges in closing the referral loop [39], difficulties in obtaining mental health referrals [40], and the need for more effective interventions to improve referral appropriateness [41]. As healthcare systems continue to evolve, addressing these challenges will be crucial for maintaining the effectiveness of primary care referrals.

Patient perceptions of care, emotional responses, and behavioral factors play a crucial role in shaping overall satisfaction. Research suggests that feelings of safety, trust in medical staff, and the perception of being heard and respected contribute significantly to positive patient experiences [42,43,44]. In our study, respondents who reported higher satisfaction levels also frequently highlighted aspects related to emotional comfort, such as clear communication, attentiveness of medical staff, and spiritual support.

Furthermore, the willingness to return for future medical services may be linked to patient confidence in the hospital’s ability to provide not only quality care but also a psychologically supportive environment. Patients who experienced anxiety or distress during hospitalization—especially those unaware of complaint submission processes—may have had lower satisfaction scores [45]. These psychological dimensions underline the importance of holistic care approaches that address both medical and emotional needs.

These aspects are closely related to the presence of medical staff and family accompanying patients from the admission office to the ward, which significantly increased over the years, with 85.2% of patients reporting being accompanied by medical staff by 2022. This improvement reflects a positive shift in hospital care, enhancing patient comfort and reassurance during the admission process. Similarly, the presence of family members accompanying patients also increased from 49.4% in 2019 to 79.5% in 2022, demonstrating the growing involvement of relatives in the hospitalization process, which could be due to heightened awareness of the importance of family support in improving patient outcomes. Gheshlaghi et al. emphasized in a new study that family presence significantly reduces the anxiety level in patients, especially during procedures happening in the emergency room [46]. Also, family involvement in cancer care can improve patient outcomes and treatment effectiveness. Family members play a central role in supporting patients through diagnosis, treatment, and recovery [47]. Their involvement can lead to better addiction treatment outcomes for patients with substance use disorders [48]. In the context of dementia care, family caregivers face major challenges, but family-based therapy interventions can enhance their ability to provide care, potentially improving outcomes for dementia patients [49]. For patients undergoing chemotherapy, a cognitive behavioral intervention involving both patients and family caregivers led to reduced symptom severity among patients in the intervention group [50]. Interestingly, in intensive care settings, the impact extends beyond just the patient. The intensive care experience has both psychological and physiological effects on family members, which in turn can influence patient recovery [51]. This highlights the interconnected nature of patient and family well-being. Family presence and involvement generally have positive effects on patient outcomes across various medical conditions. However, it is important to note that caregiving can be stressful for family members, potentially impacting their own health [52]. Therefore, healthcare systems should consider adopting family-centric models of care [48] and provide support to family caregivers to optimize both patient and family outcomes.

When patients were asked about the quality of medical care provided, both by doctors and nurses, the majority consistently rated it highly. The percentage of patients giving top marks (5/5) to the care received from doctors remained around 82% for all years, with similar levels of satisfaction for nursing care (81%). These high ratings suggest that the hospital is succeeding in maintaining quality medical care across different specialties. 

However, there was a significant gap in patient awareness regarding their rights, with a notable portion of patients in all years indicating that they were not informed about their rights and obligations. While the percentage of informed patients increased over the years, with a peak of 53.8% in 2022, this area remains a challenge for the hospital. These findings align with ongoing worldwide trends. Zulfikar et al. [53] showed in their study that less than 25% of patients knew their rights. In 2018, Mohammed et al. [54] reported the same concerning results, in their study conducted with patients at Minia University Hospital, Minia, Egypt. It is crucial to highlight that the lack of patient awareness regarding their rights and responsibilities is particularly prevalent in developing countries. In these settings, limited access to medical education, healthcare resources, and information dissemination can significantly hinder patients’ understanding of their entitlements and obligations within the healthcare system. A more solid approach to informing patients about their rights, possibly through pamphlets or digital means, could improve patient satisfaction and ensure greater transparency in hospital procedures. 

The issue of communication between medical staff and patients was further highlighted in questions about informing patients about their diagnosis, treatment plans, and risks of treatment. While most patients reported receiving adequate information, with over 90% in 2022 affirming that they were informed about their diagnosis and treatment plan, the awareness of the side effects of medications still showed room for improvement. Only 73.3% of patients in 2022 indicated that they were informed about the side effects of medications, suggesting that there is a need for more detailed communication regarding potential side effects, especially for patients undergoing complex treatments or receiving multiple medications. In a very recent study, Bahari et al. emphasized that in their study conducted in Ardabil University of Medical Sciences, Iran, with 378 patients involved, the nurses’ communication skills were graded as moderate, even though none of the patients rated them as poor. This indicates that communication between medical staff and patients might be a general problem [55], and the same conclusion was drawn in the study of Katsaliaki in 2022 [56]. Physician expertise, including trust and good communication skills, is highly valued by patients and contributes significantly to their satisfaction with hospitalization [5]. 

Regarding medication, the data suggest that the majority of patients did not purchase medications during their hospitalization, though a significant portion (87%) still reported purchasing medications. The information on side effects and prescribed medications indicates that patients may benefit from more tailored communication about the potential risks and benefits of their treatment plans. 

We examined how key trends evolved over time and the extent to which external factors, particularly the COVID-19 pandemic, influenced patient experiences. The data spanning 2019–2022 reveal significant shifts in hospital services and patient perceptions. Notably, the highest rate of hospitalizations occurred in 2020, accounting for 36% of total admissions. This peak coincided with the height of the pandemic, which likely affected satisfaction levels due to increased patient volume, strained medical resources, and the enforcement of strict hospital policies.

Despite these challenges, overall satisfaction remained high throughout the study period, though minor fluctuations were observed in specific service areas. For instance, hospital cleanliness and ward conditions received slightly higher ratings in 2020 and 2021, potentially reflecting the impact of stricter hygiene protocols implemented in response to the pandemic. At the same time, certain aspects of patient experience, such as emotional support and continuity of care, may have been negatively affected by limitations on family visits, procedural delays, and increased staff workload.

Beyond logistical and operational factors, the pandemic also influenced patient psychology and behavior. Increased concern for safety and hygiene heightened patient expectations regarding hospital cleanliness and infection control measures. Additionally, the stress and uncertainty associated with COVID-19 may have amplified the importance of clear communication, emotional reassurance, and perceived trust in medical staff. These psychological and behavioral responses likely shaped patient satisfaction scores over time, highlighting the need for healthcare institutions to address not only the quality of medical services but also the emotional and psychological well-being of patients.

By incorporating these longitudinal insights, our study provides a more comprehensive understanding of the factors influencing patient satisfaction over time. Recognizing the interplay between external circumstances, hospital policies, and patient perceptions is essential for designing adaptive healthcare strategies that ensure both high-quality medical care and a supportive hospital environment.

Several strengths of hospitals operating within a decentralized system can be identified, including the quality of medical care provided by physicians and nurses, the cleanliness of the hospital and its wards, and the elimination of the need for patients to procure medication externally. It has been empirically demonstrated that the quality of medical services is directly correlated with the professional training level of medical staff, as well as the mental and physical well-being of physicians and nurses [57]. The significant role of investments in physical healthcare resources (such as facilities, technology, and equipment) and human resources for continuous development has been underscored by recent global health policy initiatives [58]. These initiatives are characterized by concurrent proposals concerning various areas of sustainable development, clearly emphasizing the imperative for consolidation within the healthcare sector [59,60,61,62].

Overall, the hospital has shown consistent improvements in many key areas of patient satisfaction, particularly in the attitude of medical and nursing staff, the quality of medical care, and patient support during hospitalization. However, areas such as patient rights communication, awareness of medication side effects, and fall risk prevention require continued attention to ensure that the hospital provides comprehensive, patient-centered care.

### Limitations of the Study and Future Research

This study was designed to evaluate patients’ perceptions of the quality of care provided by medical staff, the services offered by the hospital, and the overall impression of the hospital, rather than to conduct inter-departmental comparisons. In addition, a key objective was to identify the most effective rating scale through a comparative analysis of patient satisfaction questionnaires. It is important to note that the data available for this research do not encompass all patients admitted and discharged during the study period, which may limit the generalizability of the findings. Furthermore, the retrospective design and reliance on self-reported data may have introduced biases such as recall bias and social desirability bias.

Future research should address these limitations by employing prospective study designs that capture a more comprehensive patient population. Additional studies could also explore inter-departmental variations in patient satisfaction to provide a more granular understanding of service quality across different hospital units. Moreover, further investigation into the effectiveness of various rating scales in capturing patient satisfaction would be beneficial. Future work should also examine the impact of targeted interventions—such as improved communication of patient rights and streamlined complaint procedures—on overall patient satisfaction and explore the underlying factors contributing to gender-based disparities in specialty utilization. These avenues of research will be critical for developing more effective, patient-centered strategies that enhance healthcare quality and outcomes.

## 5. Conclusions

The study results indicate high overall patient satisfaction at the Bihor County Emergency Clinical Hospital, especially in cleanliness, medical care quality, and patient information. Specifically, 62% of patients rated their experience as excellent, and over 90% were satisfied with the information on diagnosis and treatment. However, improvements are needed in informing patients about complaint processes, as only 67% were aware. Less than 54% knew their rights through official channels. While patients were satisfied with care from doctors and nurses, gender disparities emerged in specialty utilization, with more male admissions in cardiology and neurosurgery. These findings highlight the need for better communication of patient rights and complaints to enhance transparency and trust. Additionally, the gender-based differences in specialty use suggest that further research is needed to understand factors like referral patterns or sociocultural influences.

## Figures and Tables

**Table 1:** Patient satisfaction survey results for hospital services.

Characteristic	N = 12,802
Gender	
Female	7492 (59%)
Male	5310 (41%)
Education Level	
High school	6416 (50%)
Middle school (8 grades)	2194 (17%)
No response	302 (2.4%)
Primary	770 (6.0%)
University	3120 (24%)
Age	54 (40, 65)
Residence	
No Response	2068 (16%)
Rural	5168 (40%)
Urban	5566 (43%)
Gender	
Female	7492 (59%)
Male	5310 (41%)
Year	
2019	3876 (30%)
2020	4598 (36%)
2021	3712 (29%)
2022	616 (4.8%)

Data are presented as N (%).

**Table 2:** Yearly comparison of patient experience in accessibility and admission.

	2019 (N = 3876)	2020 (N = 4598)	2021 (N = 3712)	2022 (N = 616)	Total (N = 12,802)	*p* Value
How did you seek hospitalization in our hospital?						<0.001 ^1^
Arrived by ambulance	826.0 (21.3%)	1328.0 (28.9%)	1022.0 (27.5%)	200.0 (32.5%)	3376.0 (26.4%)	
No response	82.0 (2.1%)	100.0 (2.2%)	186.0 (5.0%)	28.0 (4.5%)	396.0 (3.1%)	
Others situation	346.0 (8.9%)	396.0 (8.6%)	310.0 (8.4%)	50.0 (8.1%)	1102.0 (8.6%)	
Presented directly to the emergency room	1166.0 (30.1%)	1746.0 (38.0%)	1492.0 (40.2%)	196.0 (31.8%)	4600.0 (35.9%)	
Referred by the family doctor	1074.0 (27.7%)	686.0 (14.9%)	414.0 (11.2%)	104.0 (16.9%)	2278.0 (17.8%)	
Referred by the outpatient doctor	382.0 (9.9%)	342.0 (7.4%)	288.0 (7.8%)	38.0 (6.2%)	1050.0 (8.2%)	
Were you accompanied by medical staff from the admission office to the ward?						<0.001 ^1^
No	594.0 (15.3%)	284.0 (6.2%)	108.0 (2.9%)	28.0 (4.5%)	1014.0 (7.9%)	
No response	268.0 (6.9%)	356.0 (7.7%)	210.0 (5.7%)	44.0 (7.1%)	878.0 (6.9%)	
Yes	3014.0 (77.8%)	3958.0 (86.1%)	3394.0 (91.4%)	544.0 (88.3%)	10910.0 (85.2%)	
Were you accompanied by relatives from the admission office to the ward?						<0.001 ^1^
No	1608.0 (41.5%)	2928.0 (63.7%)	2844.0 (76.6%)	490.0 (79.5%)	7870.0 (61.5%)	
No response	352.0 (9.1%)	454.0 (9.9%)	292.0 (7.9%)	42.0 (6.8%)	1140.0 (8.9%)	
Yes	1916.0 (49.4%)	1216.0 (26.4%)	576.0 (15.5%)	84.0 (13.6%)	3792.0 (29.6%)	
Were you accompanied by assigned staff during hospital movements (e.g., for tests)?						<0.001 ^1^
No	412.0 (10.6%)	326.0 (7.1%)	192.0 (5.2%)	38.0 (6.2%)	968.0 (7.6%)	
No response	292.0 (7.5%)	308.0 (6.7%)	188.0 (5.1%)	28.0 (4.5%)	816.0 (6.4%)	
Yes	3172.0 (81.8%)	3964.0 (86.2%)	3332.0 (89.8%)	550.0 (89.3%)	11,018.0 (86.1%)	

^1.→^Chi-square tests were used for statistical analysis, and N (%) values are presented in the table.

**Table 3:** Quality of medical care across years.

	2019 (N = 3876)	2020 (N = 4598)	2021 (N = 3712)	2022 (N = 616)	Total (N = 12,802)	*p* Value
Do you know the identity of the medical staff involved in providing medical services?						<0.001 ^1^
No	1302.0 (33.6%)	1614.0 (35.1%)	1318.0 (35.5%)	252.0 (40.9%)	4486.0 (35.0%)	
No response	344.0 (8.9%)	456.0 (9.9%)	362.0 (9.8%)	32.0 (5.2%)	1194.0 (9.3%)	
Yes	2230.0 (57.5%)	2528.0 (55.0%)	2032.0 (54.7%)	332.0 (53.9%)	7122.0 (55.6%)	
How do you rate the attitude of the hospital staff?						<0.001 ^1^
1	716.0 (18.5%)	56.0 (1.2%)	44.0 (1.2%)	4.0 (0.6%)	820.0 (6.4%)	
2	24.0 (0.6%)	16.0 (0.3%)	14.0 (0.4%)	2.0 (0.3%)	56.0 (0.4%)	
3	218.0 (5.6%)	124.0 (2.7%)	124.0 (3.3%)	16.0 (2.6%)	482.0 (3.8%)	
4	508.0 (13.1%)	958.0 (20.8%)	632.0 (17.0%)	108.0 (17.5%)	2206.0 (17.2%)	
5	2286.0 (59.0%)	3264.0 (71.0%)	2762.0 (74.4%)	462.0 (75.0%)	8774.0 (68.5%)	
No response	124.0 (3.2%)	180.0 (3.9%)	136.0 (3.7%)	24.0 (3.9%)	464.0 (3.6%)	
Quality of medical care provided by your doctor						<0.001 ^1^
1	100.0 (2.6%)	40.0 (0.9%)	28.0 (0.8%)	4.0 (0.6%)	172.0 (1.3%)	
2	18.0 (0.5%)	10.0 (0.2%)	12.0 (0.3%)	2.0 (0.3%)	42.0 (0.3%)	
3	62.0 (1.6%)	96.0 (2.1%)	54.0 (1.5%)	26.0 (4.2%)	238.0 (1.9%)	
4	370.0 (9.5%)	610.0 (13.3%)	506.0 (13.6%)	76.0 (12.3%)	1562.0 (12.2%)	
5	3228.0 (83.3%)	3756.0 (81.7%)	3044.0 (82.0%)	500.0 (81.2%)	10,528.0 (82.2%)	
No response	98.0 (2.5%)	86.0 (1.9%)	68.0 (1.8%)	8.0 (1.3%)	260.0 (2.0%)	
Quality of medical care provided by your nurses						<0.001 ^1^
1	86.0 (2.2%)	32.0 (0.7%)	46.0 (1.2%)	4.0 (0.6%)	168.0 (1.3%)	
2	12.0 (0.3%)	22.0 (0.5%)	14.0 (0.4%)	4.0 (0.6%)	52.0 (0.4%)	
3	88.0 (2.3%)	104.0 (2.3%)	54.0 (1.5%)	28.0 (4.5%)	274.0 (2.1%)	
4	414.0 (10.7%)	644.0 (14.0%)	484.0 (13.0%)	66.0 (10.7%)	1608.0 (12.6%)	
5	3154.0 (81.4%)	3704.0 (80.6%)	3024.0 (81.5%)	504.0 (81.8%)	10,386.0 (81.1%)	
6	122.0 (3.1%)	92.0 (2.0%)	90.0 (2.4%)	10.0 (1.6%)	314.0 (2.5%)	
Quality of medical care provided by your orderliers						<0.001 ^1^
1	82.0 (2.1%)	36.0 (0.8%)	48.0 (1.3%)	2.0 (0.3%)	168.0 (1.3%)	
2	44.0 (1.1%)	30.0 (0.7%)	40.0 (1.1%)	4.0 (0.6%)	118.0 (0.9%)	
3	170.0 (4.4%)	118.0 (2.6%)	66.0 (1.8%)	34.0 (5.5%)	388.0 (3.0%)	
4	444.0 (11.5%)	734.0 (16.0%)	536.0 (14.4%)	84.0 (13.6%)	1798.0 (14.0%)	
5	2988.0 (77.1%)	3582.0 (77.9%)	2924.0 (78.8%)	480.0 (77.9%)	9974.0 (77.9%)	
No response	148.0 (3.8%)	98.0 (2.1%)	98.0 (2.6%)	12.0 (1.9%)	356.0 (2.8%)	

^1.→^Chi-square tests were used for statistical analysis, with N (%) values reported in the table, including the distribution of Likert scale responses (1–5).

**Table 4:** Yearly comparison of patient safety and rights.

	2019 (N = 3876)	2020 (N = 4598)	2021 (N = 3712)	2022 (N = 616)	Total (N = 12,802)	*p* Value
Were you informed about your rights and obligations in the hospital?						<0.001 ^1^
N-Miss	2.0	0.0	0.0	0.0	2.0	
No	374.0 (9.7%)	250.0 (5.4%)	216.0 (5.8%)	56.0 (9.1%)	896.0 (7.0%)	
No response	350.0 (9.0%)	344.0 (7.5%)	352.0 (9.5%)	66.0 (10.7%)	1112.0 (8.7%)	
Yes, at the admission office	2136.0 (55.1%)	2460.0 (53.5%)	1994.0 (53.7%)	300.0 (48.7%)	6890.0 (53.8%)	
Yes, only verbally	1014.0 (26.2%)	1544.0 (33.6%)	1150.0 (31.0%)	194.0 (31.5%)	3902.0 (30.5%)	
Were you informed about the process for submitting suggestions and complaints?						<0.001 ^1^
No	912.0 (23.5%)	926.0 (20.1%)	708.0 (19.1%)	120.0 (19.5%)	2666.0 (20.8%)	
No response	486.0 (12.5%)	538.0 (11.7%)	422.0 (11.4%)	74.0 (12.0%)	1520.0 (11.9%)	
Yes	2478.0 (63.9%)	3134.0 (68.2%)	2582.0 (69.6%)	422.0 (68.5%)	8616.0 (67.3%)	
Were you informed about the estimated discharge date?						<0.001 ^1^
No	766.0 (19.8%)	822.0 (17.9%)	706.0 (19.0%)	120.0 (19.5%)	2414.0 (18.9%)	
No response	274.0 (7.1%)	236.0 (5.1%)	172.0 (4.6%)	46.0 (7.5%)	728.0 (5.7%)	
Yes	2836.0 (73.2%)	3540.0 (77.0%)	2834.0 (76.3%)	450.0 (73.1%)	9660.0 (75.5%)	
Were you informed about the risk of falling?						<0.001 ^1^
No	576.0 (14.9%)	442.0 (9.6%)	352.0 (9.5%)	58.0 (9.4%)	1428.0 (11.2%)	
No response	370.0 (9.5%)	354.0 (7.7%)	250.0 (6.7%)	44.0 (7.1%)	1018.0 (8.0%)	
Yes	2930.0 (75.6%)	3802.0 (82.7%)	3110.0 (83.8%)	514.0 (83.4%)	10356.0 (80.9%)	
Were you informed about your diagnosis?						<0.001 ^1^
No	130.0 (3.4%)	98.0 (2.1%)	74.0 (2.0%)	14.0 (2.3%)	316.0 (2.5%)	
No response	122.0 (3.1%)	106.0 (2.3%)	74.0 (2.0%)	10.0 (1.6%)	312.0 (2.4%)	
Yes	3624.0 (93.5%)	4394.0 (95.6%)	3564.0 (96.0%)	592.0 (96.1%)	12,174.0 (95.1%)	
Did you receive information about how the disease will progress and the treatment plan?						<0.001 ^1^
No	264.0 (6.8%)	234.0 (5.1%)	174.0 (4.7%)	44.0 (7.1%)	716.0 (5.6%)	
No response	308.0 (7.9%)	314.0 (6.8%)	190.0 (5.1%)	30.0 (4.9%)	842.0 (6.6%)	
Yes	3304.0 (85.2%)	4050.0 (88.1%)	3348.0 (90.2%)	542.0 (88.0%)	11,244.0 (87.8%)	
Were you informed about the side effects of medications administered in the hospital?						<0.001 ^1^
Name the side effect (recorded in column AP—Side Effect)	734.0 (18.9%)	674.0 (14.7%)	562.0 (15.1%)	122.0 (19.8%)	2092.0 (16.3%)	
No	18.0 (0.5%)	10.0 (0.2%)	20.0 (0.5%)	12.0 (1.9%)	60.0 (0.5%)	
No response	344.0 (8.9%)	508.0 (11.0%)	372.0 (10.0%)	48.0 (7.8%)	1272.0 (9.9%)	
Yes	2780.0 (71.7%)	3406.0 (74.1%)	2758.0 (74.3%)	434.0 (70.5%)	9378.0 (73.3%)	
Can you name a medication administered to you in the hospital?						<0.001 ^1^
Yes	1552.0 (40.0%)	1534.0 (33.4%)	1126.0 (30.3%)	264.0 (42.9%)	4476.0 (35.0%)	
No	1474.0 (38.0%)	1766.0 (38.4%)	1516.0 (40.8%)	204.0 (33.1%)	4960.0 (38.7%)	
No response	850.0 (21.9%)	1298.0 (28.2%)	1070.0 (28.8%)	148.0 (24.0%)	3366.0 (26.3%)	
Did you purchase medications during your hospitalization?						0.015 ^1^
Name the purchased medication (recorded in column AR—Purchased Medication)	3414.0 (88.1%)	3990.0 (86.8%)	3202.0 (86.3%)	536.0 (87.0%)	11,142.0 (87.0%)	
No	26.0 (0.7%)	36.0 (0.8%)	48.0 (1.3%)	10.0 (1.6%)	120.0 (0.9%)	
No response	198.0 (5.1%)	242.0 (5.3%)	212.0 (5.7%)	38.0 (6.2%)	690.0 (5.4%)	
Yes	238.0 (6.1%)	330.0 (7.2%)	250.0 (6.7%)	32.0 (5.2%)	850.0 (6.6%)	
Were the vials administered opened in your presence?						0.190 ^1^
No	358.0 (9.2%)	406.0 (8.8%)	346.0 (9.3%)	74.0 (12.0%)	1184.0 (9.2%)	
No response	270.0 (7.0%)	338.0 (7.4%)	232.0 (6.2%)	46.0 (7.5%)	886.0 (6.9%)	
Not applicable	490.0 (12.6%)	588.0 (12.8%)	494.0 (13.3%)	68.0 (11.0%)	1640.0 (12.8%)	
Yes	2758.0 (71.2%)	3266.0 (71.0%)	2640.0 (71.1%)	428.0 (69.5%)	9092.0 (71.0%)	
Did the medical staff use disposable gloves at each contact with you?						0.002 ^1^
1	3652.0 (94.2%)	4408.0 (95.9%)	3554.0 (95.7%)	588.0 (95.5%)	12,202.0 (95.3%)	
2	76.0 (2.0%)	56.0 (1.2%)	54.0 (1.5%)	4.0 (0.6%)	190.0 (1.5%)	
3	148.0 (3.8%)	134.0 (2.9%)	104.0 (2.8%)	24.0 (3.9%)	410.0 (3.2%)	
Were you operated on during your hospitalization?						0.078 ^1^
1	1742.0 (44.9%)	1908.0 (41.5%)	1634.0 (44.0%)	262.0 (42.5%)	5546.0 (43.3%)	
2	1926.0 (49.7%)	2432.0 (52.9%)	1880.0 (50.6%)	320.0 (51.9%)	6558.0 (51.2%)	
3	208.0 (5.4%)	258.0 (5.6%)	198.0 (5.3%)	34.0 (5.5%)	698.0 (5.5%)	
How do you rate postoperative care and the caregiver provided in the intensive care unit (if applicable)?						<0.001 ^1^
1	56.0 (1.4%)	24.0 (0.5%)	20.0 (0.5%)	0.0 (0.0%)	100.0 (0.8%)	
2	22.0 (0.6%)	12.0 (0.3%)	42.0 (1.1%)	2.0 (0.3%)	78.0 (0.6%)	
3	56.0 (1.4%)	74.0 (1.6%)	66.0 (1.8%)	2.0 (0.3%)	198.0 (1.5%)	
4	232.0 (6.0%)	318.0 (6.9%)	232.0 (6.2%)	26.0 (4.2%)	808.0 (6.3%)	
5	1384.0 (35.7%)	1668.0 (36.3%)	1404.0 (37.8%)	202.0 (32.8%)	4658.0 (36.4%)	
No response	2126.0 (54.9%)	2502.0 (54.4%)	1948.0 (52.5%)	384.0 (62.3%)	6960.0 (54.4%)	
Did you reward any medical staff (doctor, nurse, orderly, care giver, stretcher-bearer, etc.) with money or gifts during your hospitalization?						<0.001 ^1^
1	416.0 (10.7%)	76.0 (1.7%)	102.0 (2.7%)	2.0 (0.3%)	596.0 (4.7%)	
2	3002.0 (77.5%)	4062.0 (88.3%)	3250.0 (87.6%)	518.0 (84.1%)	10,832.0 (84.6%)	
3	458.0 (11.8%)	460.0 (10.0%)	360.0 (9.7%)	96.0 (15.6%)	1374.0 (10.7%)	
If the answer to the previous question was YES, please specify the category of medical staff:						<0.001 ^1^
Doctor	100.0 (2.6%)	46.0 (1.0%)	60.0 (1.6%)	4.0 (0.6%)	210.0 (1.6%)	
Not applicable/No response	2650.0 (68.4%)	4432.0 (96.4%)	3478.0 (93.7%)	612.0 (99.4%)	11,172.0 (87.3%)	
Nurse	1008.0 (26.0%)	14.0 (0.3%)	24.0 (0.6%)	0.0 (0.0%)	1046.0 (8.2%)	
Orderly	108.0 (2.8%)	12.0 (0.3%)	44.0 (1.2%)	0.0 (0.0%)	164.0 (1.3%)	
Others	6.0 (0.2%)	86.0 (1.9%)	78.0 (2.1%)	0.0 (0.0%)	170.0 (1.3%)	
Stretcher-bearer	4.0 (0.1%)	8.0 (0.2%)	28.0 (0.8%)	0.0 (0.0%)	40.0 (0.3%)	
How do you rate the quality of hospital food?						<0.001 ^1^
1	162.0 (4.2%)	136.0 (3.0%)	96.0 (2.6%)	8.0 (1.3%)	402.0 (3.1%)	
2	206.0 (5.3%)	232.0 (5.0%)	166.0 (4.5%)	12.0 (1.9%)	616.0 (4.8%)	
3	606.0 (15.6%)	956.0 (20.8%)	596.0 (16.1%)	82.0 (13.3%)	2240.0 (17.5%)	
4	1074.0 (27.7%)	1542.0 (33.5%)	1246.0 (33.6%)	244.0 (39.6%)	4106.0 (32.1%)	
5	1430.0 (36.9%)	1394.0 (30.3%)	1324.0 (35.7%)	224.0 (36.4%)	4372.0 (34.2%)	
No response	398.0 (10.3%)	338.0 (7.4%)	284.0 (7.7%)	46.0 (7.5%)	1066.0 (8.3%)	
Are you satisfied with the ward conditions (equipment, facilities)?						<0.001 ^1^
1	84.0 (2.2%)	34.0 (0.7%)	38.0 (1.0%)	2.0 (0.3%)	158.0 (1.2%)	
2	108.0 (2.8%)	60.0 (1.3%)	64.0 (1.7%)	8.0 (1.3%)	240.0 (1.9%)	
3	296.0 (7.6%)	400.0 (8.7%)	306.0 (8.2%)	34.0 (5.5%)	1036.0 (8.1%)	
4	970.0 (25.0%)	1448.0 (31.5%)	1184.0 (31.9%)	188.0 (30.5%)	3790.0 (29.6%)	
5	2244.0 (57.9%)	2498.0 (54.3%)	1986.0 (53.5%)	358.0 (58.1%)	7086.0 (55.4%)	
6	174.0 (4.5%)	158.0 (3.4%)	134.0 (3.6%)	26.0 (4.2%)	492.0 (3.8%)	

^1.→^Chi-square tests were used for statistical analysis, with N (%) values reported in the table, including the distribution of Likert scale responses (1–5).

**Table 5:** Hotel conditions across years.

	2019 (N = 3876)	2020 (N = 4598)	2021 (N = 3712)	2022 (N = 616)	Total (N = 12,802)	*p* Value
How clean is your room?						<0.001 ^1^
1	82.0 (2.1%)	32.0 (0.7%)	28.0 (0.8%)	0.0 (0.0%)	142.0 (1.1%)	
2	42.0 (1.1%)	20.0 (0.4%)	24.0 (0.6%)	2.0 (0.3%)	88.0 (0.7%)	
3	148.0 (3.8%)	160.0 (3.5%)	146.0 (3.9%)	16.0 (2.6%)	470.0 (3.7%)	
4	676.0 (17.4%)	976.0 (21.2%)	836.0 (22.5%)	134.0 (21.8%)	2622.0 (20.5%)	
5	2810.0 (72.5%)	3288.0 (71.5%)	2580.0 (69.5%)	444.0 (72.1%)	9122.0 (71.3%)	
No response	118.0 (3.0%)	122.0 (2.7%)	98.0 (2.6%)	20.0 (3.2%)	358.0 (2.8%)	
How many times a day is your room cleaned?						<0.001 ^1^
As needed	2560.0 (66.0%)	3084.0 (67.1%)	2466.0 (66.4%)	434.0 (70.5%)	8544.0 (66.7%)	
No response	236.0 (6.1%)	240.0 (5.2%)	242.0 (6.5%)	60.0 (9.7%)	778.0 (6.1%)	
Once a day	132.0 (3.4%)	152.0 (3.3%)	126.0 (3.4%)	18.0 (2.9%)	428.0 (3.3%)	
Twice a day	948.0 (24.5%)	1122.0 (24.4%)	878.0 (23.7%)	104.0 (16.9%)	3052.0 (23.8%)	
What is your opinion about the quality of sanitary facilities?						<0.001 ^1^
1	122.0 (3.1%)	68.0 (1.5%)	90.0 (2.4%)	6.0 (1.0%)	286.0 (2.2%)	
2	130.0 (3.4%)	142.0 (3.1%)	132.0 (3.6%)	14.0 (2.3%)	418.0 (3.3%)	
3	414.0 (10.7%)	462.0 (10.0%)	364.0 (9.8%)	46.0 (7.5%)	1286.0 (10.0%)	
4	1056.0 (27.2%)	1574.0 (34.2%)	1300.0 (35.0%)	208.0 (33.8%)	4138.0 (32.3%)	
5	1826.0 (47.1%)	2092.0 (45.5%)	1620.0 (43.6%)	290.0 (47.1%)	5828.0 (45.5%)	
No response	328.0 (8.5%)	260.0 (5.7%)	206.0 (5.5%)	52.0 (8.4%)	846.0 (6.6%)	
Are visitation rules respected in your ward?						<0.001 ^1^
No	148.0 (3.8%)	104.0 (2.3%)	234.0 (6.3%)	28.0 (4.5%)	514.0 (4.0%)	
No response	326.0 (8.4%)	906.0 (19.7%)	968.0 (26.1%)	162.0 (26.3%)	2362.0 (18.5%)	
Yes	3402.0 (87.8%)	3588.0 (78.0%)	2510.0 (67.6%)	426.0 (69.2%)	9926.0 (77.5%)	

^1.→^Chi-square tests were used for statistical analysis, with N (%) values reported in the table, including the distribution of Likert scale responses (1–5).

**Table 6:** Yearly Comparison of patient safety, rights, and overall satisfaction.

	2019 (N = 3876)	2020 (N = 4598)	2021 (N = 3712)	2022 (N = 616)	Total (N = 12,802)	*p* Value
Have you been hospitalized in this hospital before?						<0.001 ^1^
No	1422.0 (36.7%)	1780.0 (38.7%)	1454.0 (39.2%)	226.0 (36.7%)	4882.0 (38.1%)	
No response	170.0 (4.4%)	212.0 (4.6%)	136.0 (3.7%)	54.0 (8.8%)	572.0 (4.5%)	
Yes	2284.0 (58.9%)	2606.0 (56.7%)	2122.0 (57.2%)	336.0 (54.5%)	7348.0 (57.4%)	
If you need medical services in the future, would you return here?						<0.001 ^1^
1	132.0 (3.4%)	40.0 (0.9%)	74.0 (2.0%)	12.0 (1.9%)	258.0 (2.0%)	
2	28.0 (0.7%)	28.0 (0.6%)	28.0 (0.8%)	2.0 (0.3%)	86.0 (0.7%)	
3	130.0 (3.4%)	164.0 (3.6%)	158.0 (4.3%)	26.0 (4.2%)	478.0 (3.7%)	
4	574.0 (14.8%)	914.0 (19.9%)	708.0 (19.1%)	98.0 (15.9%)	2294.0 (17.9%)	
5	2810.0 (72.5%)	3198.0 (69.6%)	2542.0 (68.5%)	436.0 (70.8%)	8986.0 (70.2%)	
No response	202.0 (5.2%)	254.0 (5.5%)	202.0 (5.4%)	42.0 (6.8%)	700.0 (5.5%)	
Were you satisfied with the spiritual assistance provided by the hospital?						<0.001 ^1^
No	94.0 (2.4%)	150.0 (3.3%)	146.0 (3.9%)	12.0 (1.9%)	402.0 (3.1%)	
No response	966.0 (24.9%)	1168.0 (25.4%)	978.0 (26.3%)	194.0 (31.5%)	3306.0 (25.8%)	
Yes	2816.0 (72.7%)	3280.0 (71.3%)	2588.0 (69.7%)	410.0 (66.6%)	9094.0 (71.0%)	
What is your overall impression of the hospital?						<0.001 ^1^
1	84.0 (2.2%)	26.0 (0.6%)	40.0 (1.1%)	4.0 (0.6%)	154.0 (1.2%)	
2	26.0 (0.7%)	26.0 (0.6%)	24.0 (0.6%)	0.0 (0.0%)	76.0 (0.6%)	
3	180.0 (4.6%)	188.0 (4.1%)	158.0 (4.3%)	26.0 (4.2%)	552.0 (4.3%)	
4	950.0 (24.5%)	1426.0 (31.0%)	1110.0 (29.9%)	176.0 (28.6%)	3662.0 (28.6%)	
5	2496.0 (64.4%)	2778.0 (60.4%)	2266.0 (61.0%)	388.0 (63.0%)	7928.0 (61.9%)	
No response	140.0 (3.6%)	154.0 (3.3%)	114.0 (3.1%)	22.0 (3.6%)	430.0 (3.4%)	

Data are presented as N (%), for the Likert-scale items, ratings range from 1 (lowest) to 5 (highest). ^1.→^Chi-square tests were used for statistical analysis, with N (%) values reported in the table, including the distribution of Likert scale responses (1–5).

## Data Availability

Data can be obtained from the corresponding author.

## Supplementary Materials

The following supporting information can be downloaded at: https://www.mdpi.com/article/10.3390/healthcare13070836/s1, Table S1: The Bonferroni correction for ANOVA.

## Appendix A

**PATIENT SATISFACTION QUESTIONNAIRE** [29]

THIS QUESTIONNAIRE IS ANONYMOUS AND CONFIDENTIAL!
YOUR RESPONSES ARE VERY IMPORTANT TO US!

Dear Patient,
In order to evaluate the medical care you received at the County Clinical Emergency Hospital Bihor and to improve the QUALITY of medical care, please kindly answer the questions below.
Answer the questions by choosing the option that best suits you.
Mark with an X in the box (□) corresponding to one of the answer options.
Mark with an X in the box (□) corresponding to the rating (1—the lowest and 5—the highest).
Please deposit the completed form in the boxes located in the ward where you were admitted.

1. In which ward were you admitted:
2. You are: □ female □ male □ no answer
3. Place of residence: □ rural □ urban □ no answer
4. Your age: ................... years □ no answer
5. Highest level of education completed:
□ primary (4 grades) □ junior high school
□ high school, vocational □ higher education □ no answer
6. How did you come to be admitted to our hospital:
□ you presented yourself directly to the emergency department □ you came by ambulance
□ you had a referral from your family doctor □ you had a referral from the outpatient doctor
□ other situation □ no answer
7. When you first entered this facility, what was your initial impression? Choose three words that best describe the situation in the reception area at that time.
□ cleanliness □ luxury □ calm □ discipline
□ disorder □ poverty □ filth □ overcrowding □ no answer
8. Did the situation in the reception area, as you have just described, affect your mood? (mark only one option)
□ it demoralized me □ it had no effect □ it boosted my morale □ no answer
9. Were you accompanied by healthcare personnel from the Admissions Office to the ward?
□ yes □ no □ no answer
10. Were you accompanied by relatives from the Admissions Office to the ward?
□ yes □ no □ no answer
11. Were you accompanied by designated personnel during your movement within the hospital (e.g., for examinations)?
□ yes □ no □ no answer
12. Do you know the identity of the medical staff involved in providing your care?
□ yes □ no □ no answer
13. How do you rate the quality of communication and the attitude of the hospital staff?
□ 1 □ 2 □ 3 □ 4 □ 5 □ no answer
14. How do you rate the quality of medical care provided by: (1—lowest, 5—highest)
a. Your doctor:
□ 1 □ 2 □ 3 □ 4 □ 5 □ no answer
b. Nursing staff:
□ 1 □ 2 □ 3 □ 4 □ 5 □ no answer
c. Nursing assistants:
□ 1 □ 2 □ 3 □ 4 □ 5 □ no answer
15. Were you informed about your rights and responsibilities in the hospital?
□ yes, at the Admissions Office □ yes, verbally only □ no □ no answer
16. Were you informed about how to submit suggestions and complaints?
□ yes □ no □ no answer
17. Were you informed about the estimated discharge date?
□ yes □ no □ no answer
18. Were you informed about the risk of falling?
□ yes □ no □ no answer
19. Were you informed about your diagnosis?
□ yes □ no □ no answer
20. Did you receive information about the progression of your illness and the therapeutic plan to be followed?
□ yes □ no □ no answer
21. Were you informed about the adverse effects of the medications administered in the hospital?
□ yes □ no □ adverse effect: ................................ □ no answer
22. Can you name a medication that was administered to you in the hospital?
□ no □ please specify the medication: ................................ □ no answer
23. During your hospitalization, did you purchase any medications?
□ yes □ no □ please specify the medication: ........................ □ no answer
24. Were the medication vials opened in front of you?
□ yes □ no □ not applicable □ no answer
25. Does the medical staff use single-use gloves for every contact with you?
□ yes □ no □ no answer
26. Were you operated on during your hospitalization?
□ yes □ no □ no answer
27. How would you rate the postoperative care and medical services provided in the Intensive Care Unit (if applicable)? (1—lowest, 5—highest)
□ 1 □ 2 □ 3 □ 4 □ 5 □ not applicable/no answer
28. Did you experience any postoperative complications?
□ yes □ no □ no answer
29. During your hospitalization, did you reward any medical staff (doctor, nurse, nursing assistant, orderly, etc.) with money or gifts?
□ yes □ no □ no answer
30. If your answer to the previous question was YES, please specify the professional category of the medical staff:
□ doctor □ nurse □ nursing assistant/caregiver
□ orderly □ others □ not applicable
31. How would you rate the quality of the food and the manner in which it is distributed in the hospital? (1—lowest, 5—highest)
□ 1 □ 2 □ 3 □ 4 □ 5 □ not applicable/no answer
32. Are you satisfied with the accommodation conditions in the ward (quality of bedding, hospital attire, equipment, facilities)?
□ 1 □ 2 □ 3 □ 4 □ 5 □ no answer
33. How would you rate the cleanliness of your ward? (1—lowest, 5—highest)
□ 1 □ 2 □ 3 □ 4 □ 5 □ no answer
34. Please specify how many times per day cleaning is performed in your ward:
□ once per day □ as needed □ twice per day □ no answer
35. What is your opinion of the hospital environment (appearance of the ward, corridor, restroom, courtyard)? (1—lowest, 5—highest)
□ 1 □ 2 □ 3 □ 4 □ 5 □ no answer
36. Is the visiting schedule respected in the ward where you were admitted?
□ yes □ no □ no answer
37. Have you been admitted to this hospital before?
□ yes □ no □ no answer
38. If you were in need of medical services, would you return here? (1—definitely not; …5—definitely yes)
□ 1 □ 2 □ 3 □ 4 □ 5 □ no answer
39. Were you satisfied with the spiritual assistance provided within the hospital?
□ yes □ no □ no answer
40. What is your overall impression of the hospital? (1—lowest, 5—highest)
□ 1 □ 2 □ 3 □ 4 □ 5 □ no answer

COMMENTS OR SUGGESTIONS to improve the quality of medical care:
...........................................................................................................................................................
...........................................................................................................................................................
