# Supplementary material for: Assessing Patient Satisfaction with Hospital Services: Perspectives from Bihor County Emergency Hospital, Romania
Source: Healthcare (Basel). 2025 Apr 7;13(7):836. doi: 10.3390/healthcare13070836 (PMC11988329; doi:10.3390/healthcare13070836)
Supplement: Supplementary file 1 [file healthcare-13-00836-s001.zip › healthcare-3504477-supplementary.pdf]

Table S1: The Bonferroni correction for ANOVA.

Yearly Comparison of Patient Experience in Accessibility and Admission

Variable	Comparison	Chi-square	Raw p-value	Bonferroni Alpha	Significant?
**1. How did you seek hospitalization in our hospital?**	2021 vs 2020	74.374	<0.000001	0.008333	Yes
	2021 vs 2019	405.6801	<0.000001	0.008333	Yes
	2021 vs 2022	30.5935	0.000011	0.008333	Yes
	2020 vs 2019	265.8253	<0.000001	0.008333	Yes
	2020 vs 2022	23.0822	0.000326	0.008333	Yes
	2019 vs 2022	74.1872	<0.000001	0.008333	Yes
**2. Were you accompanied by medical staff from the admission office to the ward?**	2021 vs 2020	66.2367	<0.000001	0.008333	Yes
	2021 vs 2019	362.6584	<0.000001	0.008333	Yes
	2021 vs 2022	7.0523	0.029418	0.008333	No
	2020 vs 2019	189.5402	<0.000001	0.008333	Yes
	2020 vs 2022	2.983	0.225031	0.008333	No
	2019 vs 2022	52.1153	<0.000001	0.008333	Yes

**3. Were you accompanied by relatives from the admission office to the ward?**	2021 vs 2020	172.47	<0.000001	0.008333	Yes
	2021 vs 2019	1066.2376	<0.000001	0.008333	Yes
	2021 vs 2022	2.5663	0.277169	0.008333	No
	2020 vs 2019	495.5665	<0.000001	0.008333	Yes
	2020 vs 2022	61.9426	<0.000001	0.008333	Yes
	2019 vs 2022	320.9213	<0.000001	0.008333	Yes
**4. Were you accompanied by assigned staff during hospital movements (e.g., tests)?**	2021 vs 2020	24.2536	0.000005	0.008333	Yes
	2021 vs 2019	103.1054	<0.000001	0.008333	Yes
	2021 vs 2022	1.2858	0.525754	0.008333	No
	2020 vs 2019	37.1033	<0.000001	0.008333	Yes
	2020 vs 2022	5.1613	0.075726	0.008333	No
	2019 vs 2022	20.7785	0.000031	0.008333	Yes

Quality of Medical Care Across Years

Variable	Comparison	Chi-square	Raw p-value	Bonferroni Alpha	Significant?
**Do you know the identity of the medical staff?**	2021 vs 2020	0.1735	0.916925	0.008333	No
**Do you know the identity of the medical staff?**	2021 vs 2019	6.2135	0.044746	0.008333	No
**Do you know the identity of the medical staff?**	2021 vs 2022	16.3738	0.000278	0.008333	Yes
**Do you know the identity of the medical staff?**	2020 vs 2019	6.2566	0.043793	0.008333	No
**Do you know the identity of the medical staff?**	2020 vs 2022	18.1791	0.000113	0.008333	Yes
**Do you know the identity of the medical staff?**	2019 vs 2022	18.0616	0.00012	0.008333	Yes
**How do you rate the attitude of hospital staff?**	2021 vs 2020	22.1474	0.000491	0.008333	Yes
**How do you rate the attitude of hospital staff?**	2021 vs 2019	678.3555	<0.000001	0.008333	Yes
**How do you rate the attitude of hospital staff?**	2021 vs 2022	2.4912	0.777819	0.008333	No
**How do you rate the attitude of hospital staff?**	2020 vs 2019	857.178	<0.000001	0.008333	Yes
**How do you rate the attitude of hospital staff?**	2020 vs 2022	5.6772	0.338907	0.008333	No
**How do you rate the attitude of hospital staff?**	2019 vs 2022	146.1691	<0.000001	0.008333	Yes
**Quality of medical care (Doctor)**	2021 vs 2020	6.010005	3.05E-01	0.008333	No
**Quality of medical care (Doctor)**	2021 vs 2019	70.673995	7.42E-14	0.008333	Yes
**Quality of medical care (Doctor)**	2021 vs 2022	23.505102	2.70E-04	0.008333	Yes
**Quality of medical care (Doctor)**	2020 vs 2019	73.812234	1.65E-14	0.008333	Yes

**Quality of medical care (Doctor)**	2020 vs 2022	12.505182	2.85E-02	0.008333	No
**Quality of medical care (Doctor)**	2019 vs 2022	35.29587	1.31E-06	0.008333	Yes
**Quality of medical care (Nurses)**	2021 vs 2020	17.290634	3.98E-03	0.008333	Yes
**Quality of medical care (Nurses)**	2021 vs 2019	29.907602	1.54E-05	0.008333	Yes
**Quality of medical care (Nurses)**	2021 vs 2022	32.892108	3.95E-06	0.008333	Yes
**Quality of medical care (Nurses)**	2020 vs 2019	66.266407	6.12E-13	0.008333	Yes
**Quality of medical care (Nurses)**	2020 vs 2022	16.350382	5.91E-03	0.008333	Yes
**Quality of medical care (Nurses)**	2019 vs 2022	23.126258	3.19E-04	0.008333	Yes
**Quality of medical care (Orderliers)**	2021 vs 2020	21.031271	7.99E-04	0.008333	Yes
**Quality of medical care (Orderliers)**	2021 vs 2019	70.894029	6.68E-14	0.008333	Yes
**Quality of medical care (Orderliers)**	2021 vs 2022	38.532937	2.95E-07	0.008333	Yes
**Quality of medical care (Orderliers)**	2020 vs 2019	104.471341	6.03E-21	0.008333	Yes
**Quality of medical care (Orderliers)**	2020 vs 2022	19.777959	1.38E-03	0.008333	Yes
**Quality of medical care (Orderliers)**	2019 vs 2022	19.213743	1.75E-03	0.008333	Yes
	2020 vs 2022	19.777959	1.38E-03	0.008333	Yes
	2019 vs 2022	19.213743	1.75E-03	0.008333	Yes

Patient Safety and Rights

Variable	Year Pair	Chi-square	Raw p-value	df	Bonferroni Alpha	Significant?
**Informed about rights/obligations**	2021 vs 2020	14.653335	2.14E-03	3	0.008333	Yes
	2021 vs 2019	54.227809	4.72E-11	4	0.008333	Yes
	2021 vs 2022	12.382461	6.18E-03	3	0.008333	Yes
	2020 vs 2019	98.545665	2.01E-20	4	0.008333	Yes

	2020 vs 2022	22.650506	4.78E-05	3	0.008333	Yes
	2019 vs 2022	11.763032	1.92E-02	4	0.008333	No
**Informed about suggestions/complaints**	2021 vs 2020	1.966667	3.74E-01	2	0.008333	No
	2021 vs 2019	28.806364	5.56E-07	2	0.008333	Yes
	2021 vs 2022	0.321457	8.52E-01	2	0.008333	No
	2020 vs 2019	18.043952	1.21E-04	2	0.008333	Yes
	2020 vs 2022	0.172159	9.18E-01	2	0.008333	No
	2019 vs 2022	5.633565	5.98E-02	2	0.008333	No
**Estimated discharge date**	2021 vs 2020	2.609448	0.271247	2	0.008333	FALSE
**Estimated discharge date**	2021 vs 2019	22.239557	0.000015	2	0.008333	TRUE
**Estimated discharge date**	2021 vs 2022	9.238572	0.00986	2	0.008333	FALSE
**Estimated discharge date**	2020 vs 2019	21.175703	0.000025	2	0.008333	TRUE
**Estimated discharge date**	2020 vs 2022	7.349181	0.02536	2	0.008333	FALSE
**Estimated discharge date**	2019 vs 2022	0.140852	0.931997	2	0.008333	FALSE
**Risk of falling**	2021 vs 2020	2.9585	0.2278085	2	0.008333	FALSE
**Risk of falling**	2021 vs 2019	79.15144	6.49E-18	2	0.008333	TRUE
**Risk of falling**	2021 vs 2022	0.139275	0.9327317	2	0.008333	FALSE
**Risk of falling**	2020 vs 2019	69.934767	6.51E-16	2	0.008333	TRUE
**Risk of falling**	2020 vs 2022	0.279411	0.8696141	2	0.008333	FALSE
**Risk of falling**	2019 vs 2022	18.7662	8.41E-05	2	0.008333	TRUE
**Informed about diagnosis**	2021 vs 2020	1.153799	0.561637	2	0.008333	FALSE
**Informed about diagnosis**	2021 vs 2019	24.095197	0.000006	2	0.008333	TRUE
**Informed about diagnosis**	2021 vs 2022	0.575975	0.749771	2	0.008333	FALSE

**Informed about diagnosis**	2020 vs 2019	18.17641	0.000113	2	0.008333	TRUE
**Informed about diagnosis**	2020 vs 2022	1.202744	0.548059	2	0.008333	FALSE
**Informed about diagnosis**	2019 vs 2022	6.525041	0.038292	2	0.008333	FALSE
**Disease progression/treatment plan**	2021 vs 2020	11.612592	0.0030086	2	0.008333	TRUE
**Disease progression/treatment plan**	2021 vs 2019	43.219675	4.12E-10	2	0.008333	TRUE
**Disease progression/treatment plan**	2021 vs 2022	6.673994	0.0355435	2	0.008333	FALSE
**Disease progression/treatment plan**	2020 vs 2019	16.14187	0.000312491	2	0.008333	TRUE
**Disease progression/treatment plan**	2020 vs 2022	7.457203	0.02402641	2	0.008333	FALSE
**Disease progression/treatment plan**	2019 vs 2022	7.237555	0.02681544	2	0.008333	FALSE
**Side effects of medications**	2021 vs 2020	8.252169	0.04107773	3	0.008333	FALSE
**Side effects of medications**	2021 vs 2019	20.579861	0.00012869	3	0.008333	TRUE
**Side effects of medications**	2021 vs 2022	25.231478	0.000013812	3	0.008333	TRUE
**Side effects of medications**	2020 vs 2019	38.52342	2.19E-08	3	0.008333	TRUE
**Side effects of medications**	2020 vs 2022	54.350152	9.45E-12	3	0.008333	TRUE
**Side effects of medications**	2019 vs 2022	18.563121	0.000336573	3	0.008333	TRUE
**Naming a medication**	2021 vs 2020	9.217177	0.00996587	2	0.008333	FALSE
**Naming a medication**	2021 vs 2019	90.061322	2.78E-20	2	0.008333	TRUE
**Naming a medication**	2021 vs 2022	38.053602	5.45E-09	2	0.008333	TRUE
**Naming a medication**	2020 vs 2019	58.76959	1.73E-13	2	0.008333	TRUE
**Naming a medication**	2020 vs 2022	21.687898	1.95E-05	2	0.008333	TRUE
**Naming a medication**	2019 vs 2022	5.526813	0.06307655	2	0.008333	FALSE

**Purchased medications**	2021 vs 2020	6.681231	0.082783	3	0.008333	FALSE
**Purchased medications**	2021 vs 2019	10.567292	0.014311	3	0.008333	FALSE
**Purchased medications**	2021 vs 2022	2.579473	0.4611	3	0.008333	FALSE
**Purchased medications**	2020 vs 2019	4.239767	0.23671	3	0.008333	FALSE
**Purchased medications**	2020 vs 2022	8.256561	0.040997	3	0.008333	FALSE
**Purchased medications**	2019 vs 2022	8.015461	0.045693	3	0.008333	FALSE
**Vials opened in your presence**	2021 vs 2020	4.606402	0.202994	3	0.008333	FALSE
**Vials opened in your presence**	2021 vs 2019	2.133226	0.54522	3	0.008333	FALSE
**Vials opened in your presence**	2021 vs 2022	7.459897	0.058598	3	0.008333	FALSE
**Vials opened in your presence**	2020 vs 2019	0.859918	0.835088	3	0.008333	FALSE
**Vials opened in your presence**	2020 vs 2022	7.493973	0.057714	3	0.008333	FALSE
**Vials opened in your presence**	2019 vs 2022	5.760996	0.123835	3	0.008333	FALSE
**Disposable gloves used**	2021 vs 2020	0.964418	0.617418	2	0.008333	FALSE
**Disposable gloves used**	2021 vs 2019	9.198148	0.010061	2	0.008333	FALSE
**Disposable gloves used**	2021 vs 2022	4.701377	0.095304	2	0.008333	FALSE
**Disposable gloves used**	2020 vs 2019	13.215755	0.00135	2	0.008333	TRUE
**Disposable gloves used**	2020 vs 2022	3.263679	0.195569	2	0.008333	FALSE
**Disposable gloves used**	2019 vs 2022	5.227047	0.073276	2	0.008333	FALSE
**Operated on during hospitalization**	2021 vs 2020	5.35168	0.068849	2	0.008333	FALSE
**Operated on during hospitalization**	2021 vs 2019	0.713035	0.70011	2	0.008333	FALSE
**Operated on during hospitalization**	2021 vs 2022	0.47659	0.78797	2	0.008333	FALSE
**Operated on during hospitalization**	2020 vs 2019	10.223721	0.006025	2	0.008333	TRUE
**Operated on during hospitalization**	2020 vs 2022	0.240099	0.886877	2	0.008333	FALSE

**Operated on during hospitalization**	2019 vs 2022	1.257404	0.533283	2	0.008333	FALSE
**Post-operative / Intensive care rating**	2021 vs 2020	28.45151	0.00003	5	0.008333	TRUE
**Post-operative / Intensive care rating**	2021 vs 2019	28.511673	0.000029	5	0.008333	TRUE
**Post-operative / Intensive care rating**	2021 vs 2022	30.588596	0.000011	5	0.008333	TRUE
**Post-operative / Intensive care rating**	2020 vs 2019	27.338753	0.000049	5	0.008333	TRUE
**Post-operative / Intensive care rating**	2020 vs 2022	23.429027	0.000279	5	0.008333	TRUE
**Post-operative / Intensive care rating**	2019 vs 2022	24.145594	0.000204	5	0.008333	TRUE
**Rewarding medical staff**	2021 vs 2020	11.836271	0.002690211	2	0.008333	TRUE
**Rewarding medical staff**	2021 vs 2019	208.47093	5.38E-46	2	0.008333	TRUE
**Rewarding medical staff**	2021 vs 2022	31.011046	1.85E-07	2	0.008333	TRUE
**Rewarding medical staff**	2020 vs 2019	334.939476	1.86E-73	2	0.008333	TRUE
**Rewarding medical staff**	2020 vs 2022	23.385928	8.35E-06	2	0.008333	TRUE
**Rewarding medical staff**	2019 vs 2022	70.986373	3.85E-16	2	0.008333	TRUE
**Category of rewarded staff**	2021 vs 2020	55.49342	1.03E-10	5	0.008333	TRUE
**Category of rewarded staff**	2021 vs 2019	1163.770579	2.07E-249	5	0.008333	TRUE
**Category of rewarded staff**	2021 vs 2022	34.003634	2.38E-06	5	0.008333	TRUE
**Category of rewarded staff**	2020 vs 2019	1532.440773	0.00E+00	5	0.008333	TRUE
**Category of rewarded staff**	2020 vs 2022	17.267088	0.004020147	5	0.008333	TRUE
**Category of rewarded staff**	2019 vs 2022	257.763581	1.19E-53	5	0.008333	TRUE
**Hospital Food Quality**	2021 vs 2020	45.314535	1.25E-08	5	0.008333	Yes
**Hospital Food Quality**	2021 vs 2019	53.635828	2.49E-10	5	0.008333	Yes
**Hospital Food Quality**	2021 vs 2022	20.0678	1.21E-03	5	0.008333	Yes
**Hospital Food Quality**	2020 vs 2019	110.599112	3.06E-22	5	0.008333	Yes

**Hospital Food Quality**	2020 vs 2022	44.079487	2.23E-08	5	0.008333	Yes
**Hospital Food Quality**	2019 vs 2022	55.861477	8.68E-11	5	0.008333	Yes
**Ward Conditions Satisfaction**	2021 vs 2020	5.378894	3.71E-01	5	0.008333	FALSE
**Ward Conditions Satisfaction**	2021 vs 2019	67.445036	3.48E-13	5	0.008333	Yes
**Ward Conditions Satisfaction**	2021 vs 2022	11.285354	4.60E-02	5	0.008333	FALSE
**Ward Conditions Satisfaction**	2020 vs 2019	98.50966	1.09E-19	5	0.008333	Yes
**Ward Conditions Satisfaction**	2020 vs 2022	10.48788	6.25E-02	5	0.008333	FALSE
**Ward Conditions Satisfaction**	2019 vs 2022	23.537093	2.66E-04	5	0.008333	Yes

Hotel Conditions

**Room Cleanliness**	2021 vs 2020	5.730217	3.33E-01	5	0.008333	FALSE
	2021 vs 2019	56.511181	6.38E-11	5	0.008333	Yes
	2021 vs 2022	9.429422	9.31E-02	5	0.008333	FALSE
	2020 vs 2019	61.146763	7.04E-12	5	0.008333	Yes
	2020 vs 2022	6.494386	2.61E-01	5	0.008333	FALSE
	2019 vs 2022	23.893665	2.28E-04	5	0.008333	Yes
**Room Cleaning Frequency**	2021 vs 2020	6.634487	8.45E-02	3	0.008333	FALSE
	2021 vs 2019	1.112343	7.74E-01	3	0.008333	FALSE
	2021 vs 2022	20.155854	1.58E-04	3	0.008333	Yes
	2020 vs 2019	3.225053	3.58E-01	3	0.008333	FALSE
	2020 vs 2022	33.520426	2.50E-07	3	0.008333	Yes
	2019 vs 2022	25.699524	1.10E-05	3	0.008333	Yes
**Sanitary Facilities Quality**	2021 vs 2020	13.137929	2.21E-02	5	0.008333	FALSE

	2021 vs 2019	70.004156	1.02E-13	5	0.008333	Yes
	2021 vs 2022	19.70529	1.42E-03	5	0.008333	Yes
	2020 vs 2019	85.559825	5.74E-17	5	0.008333	Yes
	2020 vs 2022	13.277754	2.09E-02	5	0.008333	FALSE
	2019 vs 2022	24.145475	2.04E-04	5	0.008333	Yes
**Visitation Rules**	2021 vs 2020	149.858785	2.87E-33	2	0.008333	Yes
	2021 vs 2019	469.139753	1.34E-102	2	0.008333	Yes
	2021 vs 2022	2.89253	2.35E-01	2	0.008333	FALSE
	2020 vs 2019	225.807363	9.26E-50	2	0.008333	Yes
	2020 vs 2022	28.284323	7.21E-07	2	0.008333	Yes
	2019 vs 2022	178.885388	1.43E-39	2	0.008333	Yes
	Patient Safety, Rights, and Overall Satisfaction					
**Previous Hospitalization**	2021 vs 2020	4.594532	1.01E-01	2	0.008333	FALSE
	2021 vs 2019	6.548766	3.78E-02	2	0.008333	FALSE
	2021 vs 2022	32.809446	7.51E-08	2	0.008333	Yes
	2020 vs 2019	4.363286	1.13E-01	2	0.008333	FALSE
	2020 vs 2022	19.40331	6.12E-05	2	0.008333	Yes
	2019 vs 2022	22.200461	1.51E-05	2	0.008333	Yes
**Future Return**	2021 vs 2020	23.114907	3.21E-04	5	0.008333	Yes
	2021 vs 2019	42.95411	3.78E-08	5	0.008333	Yes
	2021 vs 2022	6.435367	2.66E-01	5	0.008333	FALSE
	2020 vs 2019	101.034276	3.20E-20	5	0.008333	Yes
	2020 vs 2022	13.860529	1.65E-02	5	0.008333	FALSE

	2019 vs 2022	9.104112	1.05E-01	5	0.008333	FALSE
**Spiritual Assistance**	2021 vs 2020	4.064228	0.131058	2	0.008333	FALSE
	2021 vs 2019	17.423877	0.000165	2	0.008333	Yes
	2021 vs 2022	11.633002	0.002978	2	0.008333	Yes
	2020 vs 2019	5.81748	0.054544	2	0.008333	FALSE
	2020 vs 2022	12.487005	0.001943	2	0.008333	Yes
	2019 vs 2022	12.148173	0.002302	2	0.008333	Yes
**Overall Impression**	2021 vs 2020	8.601607	0.126049	5	0.008333	FALSE
	2021 vs 2019	39.796281	1.64E-07	5	0.008333	Yes
	2021 vs 2022	5.998326	0.306382	5	0.008333	FALSE
	2020 vs 2019	80.932991	5.35E-16	5	0.008333	Yes
	2020 vs 2022	5.2972	0.380696	5	0.008333	FALSE
	2019 vs 2022	14.25526	0.014067	5	0.008333	FALSE
